# Supplementary material for: Anterior cingulate cortex provides the neural substrates for feedback-driven iteration of decision and value representation
Source: Nat Commun. 2024 Jul 17;15:6020. doi: 10.1038/s41467-024-50388-9 (PMC11255269; doi:10.1038/s41467-024-50388-9)
Supplement: Supplementary file 1 — Supplementary Information [file 41467_2024_50388_MOESM1_ESM.pdf]

Anterior Cingulate Cortex Provides the Neural Substrates for Feedback-Driven Iteration of Decision and Value Representation

Wenqi Chen[1], Jiejunyi Liang[2], Qiyun Wu[2], Yunyun Han[1]✉

[1]Department of Neurobiology, School of Basic Medicine and Tongji Medical College, Huazhong University of Science and Technology, Wuhan 430030, PR China;

[2]State Key Laboratory of Intelligent Manufacturing Equipment and Technology, Huazhong University of Science and Technology, Wuhan, 430074, China;

These authors contributed equally: Wenqi Chen, Jiejunyi Liang;

✉e-mail: yhan@hust.edu.cn

Supplementary Fig. 1 Training of go/no-go discrimination tasks and imaging GCaMP6f in ACC neurons.

Supplementary Fig. 2 Overall population response during all four task sessions

Supplementary Fig. 3 ACC activity in the stimulus window is not determined by licking

Supplementary Fig. 4 ACC response to the go and no-go stimulus across T1-T3 phases in the Uncertain session

Supplementary Fig. 5 The anticipatory and consumption licking rates are stable throughout the Uncertain session

Supplementary Fig. 6 Outcome monitoring neurons detect the unexpected reward

Supplementary Fig. 7 The difference of outcome evoked activity in the Hit and RO trials emerges with time in the Uncertain session

Supplementary Fig. 8 Go cue evoked response in the ACC neurons remains low in the Reversal session

Supplementary Fig. 9 Value representation is consistent throughout the Re-stable session

Supplementary Fig. 10 ACC activity in the stimulus and response window correlated well with the Q-value and prediction error estimated by the SARSA model

Supplementary Fig. 11 Optogenetic inhibition of ACC delayed decision switch in the Reversal session

Supplementary Fig. 12. Optogenetic activation of ACC neurons triggers licking response

Supplementary Table 1: Statistics table

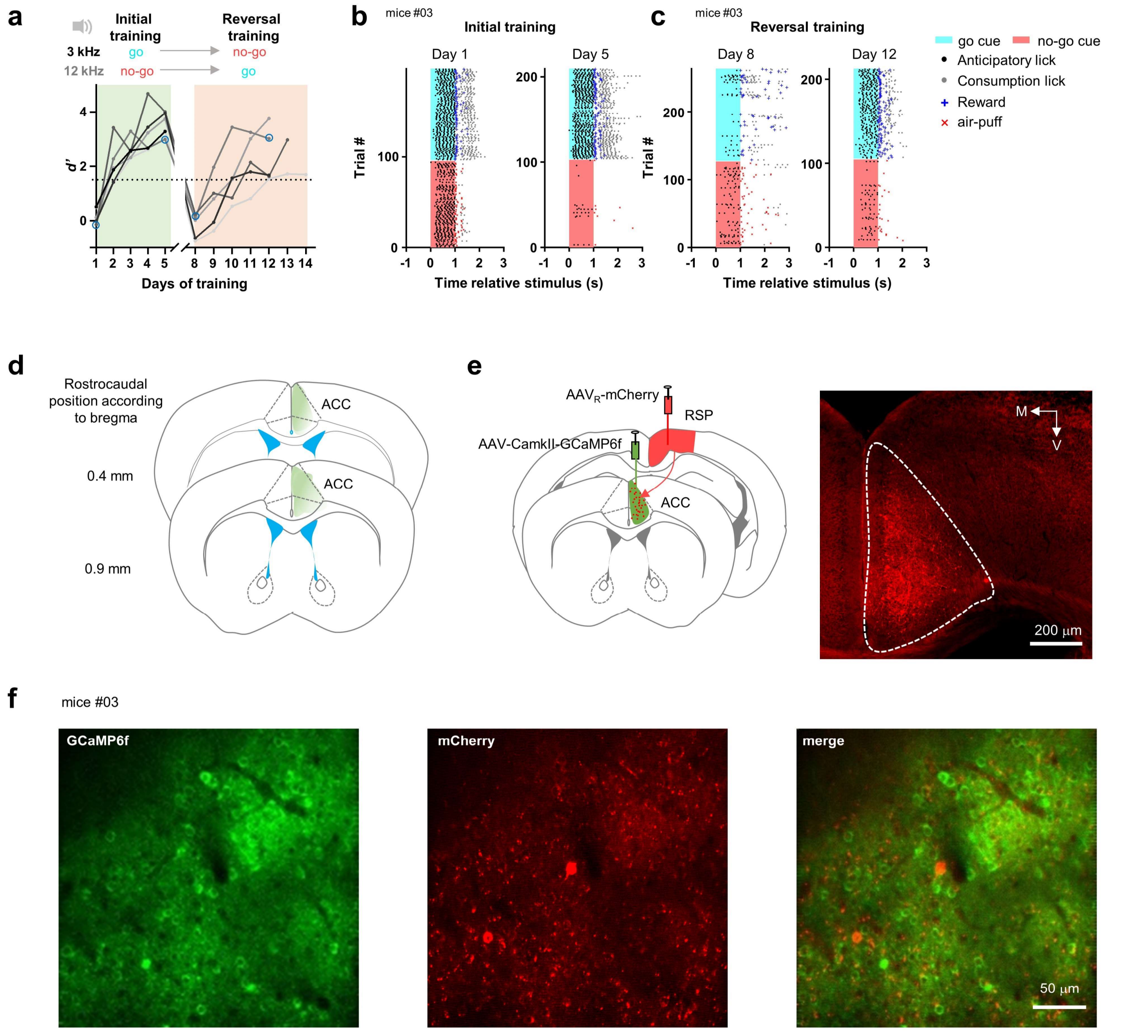

Supplementary Fig. 1 Training of go/no-go discrimination tasks and imaging GCaMP6f in ACC neurons. a Task performance (discrimination index, *d′*) of individual mice during the initial training and reversal learning process. The background color indicates the reversal of the stimulus-reward contingencies. n = 6 mice in the initial training and n = 5 mice in the reversal training. One mouse was removed when switching to reversal training due to cranial window infection. **b-c** Licking performance of an example mouse on different days of training sessions (indicated by blue circles in **a**). Trials are sorted into the go (blue shading) and no-go (red shading) trials. Dark dots: anticipatory lick (licking during stimulus window); gray dots: consumption lick (licking after reward or air-puff delivery). Plus and cross symbols represent reward and air-puff delivery in Hit and FA trials, respectively. **d** The scheme of virus-mediated expression of GCaMP6f in excitatory

neurons in the ACC (coronal view). **e** ACC boundary is identified with retrograde labeling from the retrosplenial cortex (RSP) with $AAV_{2/R}$-mCherry (left). A representative image of retrogradely labeled neurons in ACC (right) is shown in the coronal section. The white dashed line marks the boundary of ACC. **f** An example *in vivo* two-photon microscopic image of ACC neurons expressing GCaMP6f (left) and mCherry (middle, retrogradely labeled from RSP).

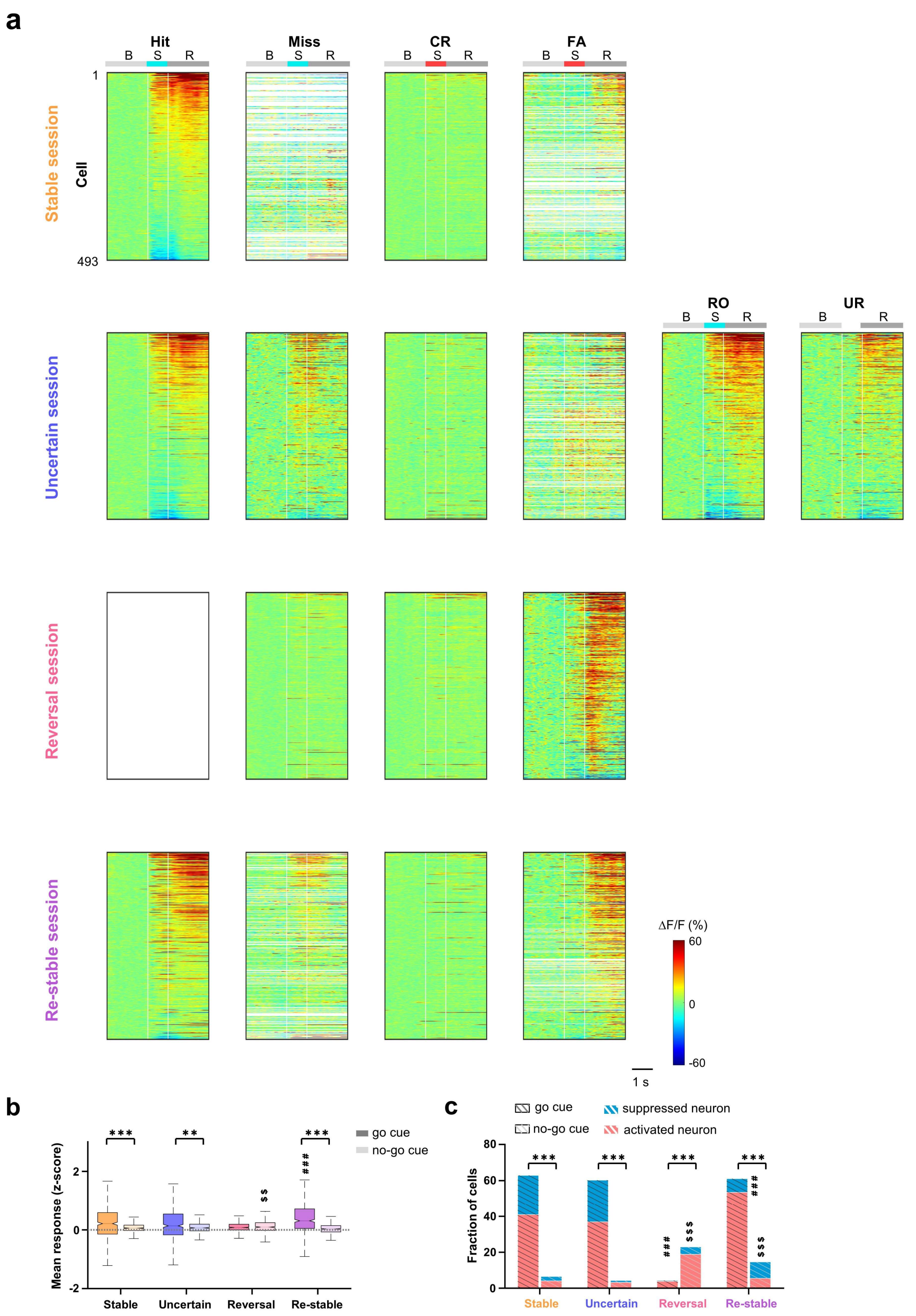

Supplementary Fig. 2 Overall population response during all four task sessions. a Trial-averaged response of recorded ACC neurons during the Hit, Miss, CR (correct rejection), FA (false alarm), RO (reward omission), and UR (un-cued reward) trials in all four task sessions (n = 493 neurons from 5 mice). Each row represents one neuron. Neurons

are sorted in the same order in all subgraphs. Vertical White lines indicate the onset of the stimulus and response windows. Horizontal whitespace indicates the lack of certain type of trials (the number of trials of one type of decision ≤ 2 times) in the recorded session. **b** Mean population response to go and no-go stimulus in four task sessions (n = 493 neurons from 5 mice). The symbol * denotes a significant difference between go and no-go responses in the same session (two-sided Wilcoxon signed-rank test). The symbol # denotes a significant difference in go response between the Stable session and the other sessions; the symbol $ represents a significant difference in no-go response between the Stable session and the other sessions (two-sided Friedman test with post-hoc Bonferroni comparisons, comparing each session with the Stable session). **c** The proportion of ACC neurons with significant activated and suppressed responses during the stimulus window in four task sessions (n = 493 neurons from 5 mice). The symbol * denotes a significant difference between go and no-go responses in the same session (two-sided chi-square test). The symbol # denotes a significant difference in go response between the Stable and the other sessions, the symbol $ represents a significant difference in no-go response between the Stable and the other sessions (two-sided chi-square test with post-hoc Bonferroni comparisons, comparing each session to the Stable session). $^{**}P < 10^{-2}$, $^{***}P < 10^{-3}$, $^{\#\#\#}P < 10^{-3}$, $^{\$\$}P < 10^{-2}$, $^{\$\$\$}P < 10^{-3}$. In box plot: center line, median; box limits, upper and lower quartiles; whiskers, 1.5 × interquartile range. Statistical details are presented in Supplementary Table 1. Source data are provided as a Source data file.

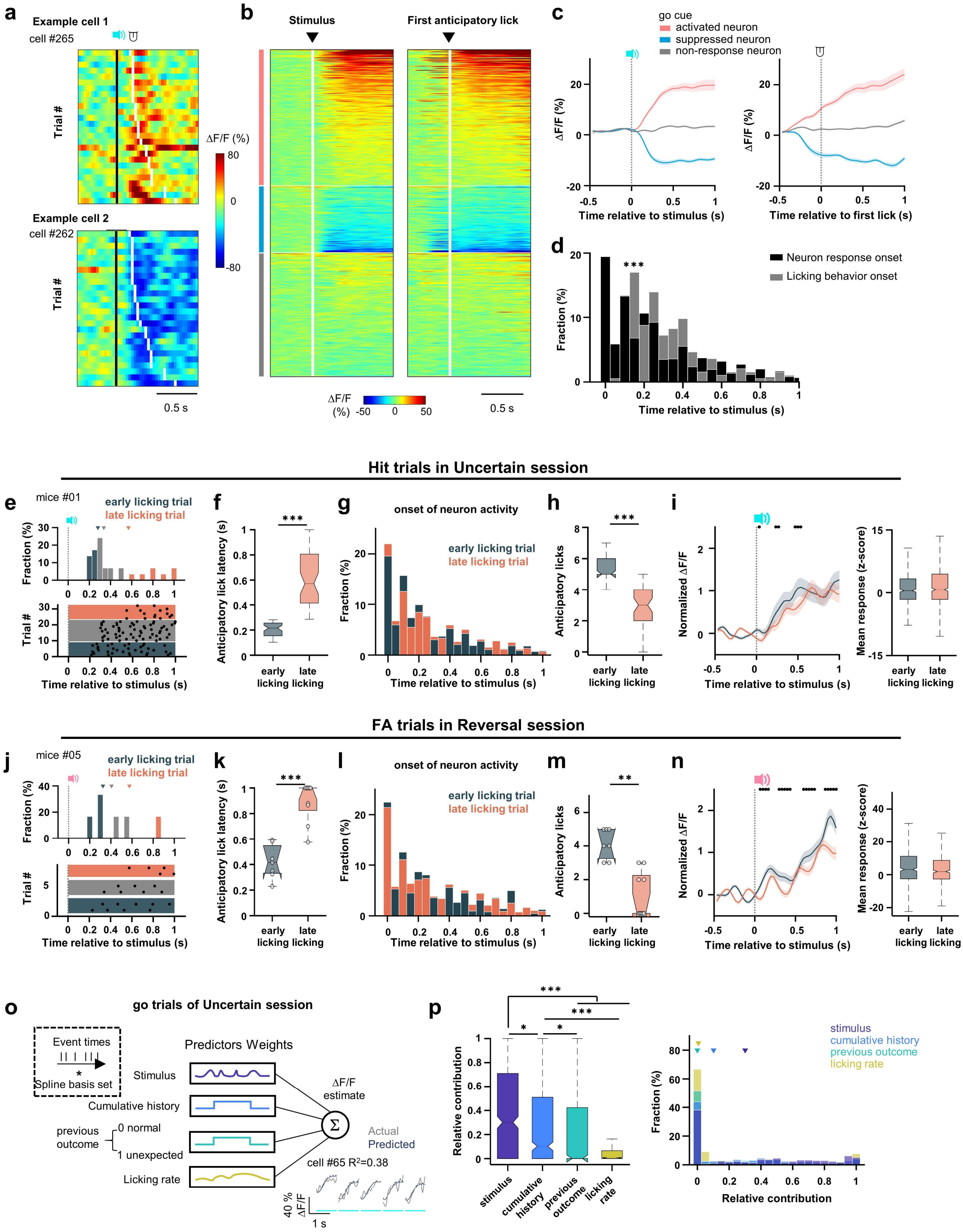

Supplementary Fig. 3 ACC activity in the stimulus window is not determined by licking. a The single-trial activity of two example neurons in Hit trials. **b** Heat maps of

trial-averaged response of all recorded neurons during Hit trials which aligned to the onset of the stimulus or aligned to the first anticipatory lick. **c** Population-averaged Ca^{2+} traces of neurons aligned to the onset of the stimulus and the first anticipatory lick (mean ± s.e.m.). **d** The histogram of neural response and the first anticipatory lick latency relative to the onsets of stimulus (n = 194 trials from 6 mice, two-sided Wilcoxon rank-sum test). **a-d** data from the Hit trials in the Stable session. **e** Top: histogram showing the distribution of anticipatory licking onset time of an example mouse of Uncertain session. All Hit trials were divided into early (blue) and late (red) lick trials according to the upper and lower quartiles of the distribution. Bottom: lick raster of the same mouse, sorted by onset times of the first lick. **f-h** Anticipatory lick latency (**f**), the histogram of neural response latency (**g**), the anticipatory lick number (**h**) of early and late licking trials (n = 49/49 trial, two-sided Wilcoxon rank-sum test). **i** Left, average population responses to go stimulus of early (blue) and late licking trials (red, mean ± s.e.m.). Right: mean population response of different trials (n = 549 neurons from 6 mice, two-sided Wilcoxon signed-rank test). **j-n** Similar to **e-i**, but the analysis for the FA trials of Reversal session. **o** Schematic of the GLM model. Inset: predicted and actual ΔF/F signal for an example neuron. **p** Left: relative contribution of each variable (n = 549 neurons from 6 mice, two-sided Kruskal-Wallis test with post hoc Bonferroni multiple comparisons). Right: the distribution of the relative contribution of each variable. *$P < 0.05$, **$P < 10^{-2}$, ***$P < 10^{-3}$. In box plot: center line, median; box limits, upper and lower quartiles; whiskers,1.5×interquartile range. Statistical details are presented in Supplementary Table 1. Source data are provided as a Source data file.

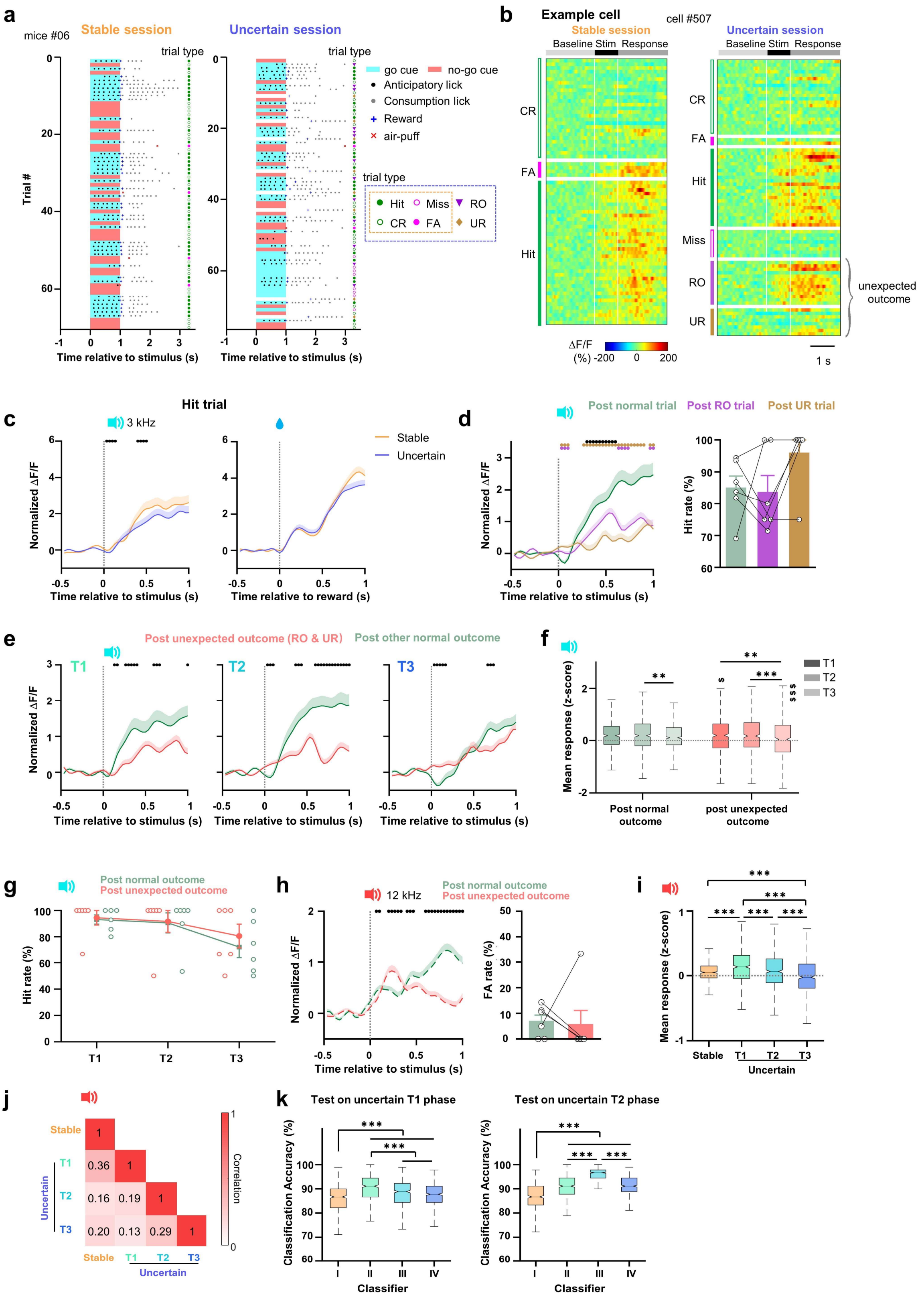

Supplementary Fig. 4 ACC response to the go and no-go stimulus across T1-T3

phases in the Uncertain session. a Licking performance and trial outcomes of an example mouse in the Stable and Uncertain sessions. **b** Single-trial activity of an example neuron. **c** Population-averaged responses to the go stimulus (left) and sucrose reward (right) in all Hit trials (n = 549 neurons from 6 mice). Dots indicate the time segments when two traces are different ($P < 0.05$, two-sided Wilcoxon rank-sum test). **d** Left, Population-averaged responses to the go stimulus following normal trials, RO trials, and UR trials. Dots indicate the time segments when traces are different ($P < 0.05$, two-sided Kruskal-Wallis test with post-hoc Bonferroni comparisons). Purple dots: post normal trials vs. post RO trials; brown dots: post normal trials vs. post UR trials; black dots: post RO trials vs. post UR trials. Right, hit rate of go trials following different outcomes. **e** Trial-averaged responses to go stimulus in trials following unexpected outcomes and following other normal outcomes in the T1-T3 phases. **f** Mean response to go stimulus flowing different outcome in the T1-T3 phases (n = 549 neurons from 6 mice. Symbol *: differences between the trials following the same outcomes in different phases (two-sided Friedman test with post-hoc Bonferroni comparisons), $: differences between the trials following different outcomes in the same phase (two-sided Wilcoxon signed-rank test). **g** The Hit rate of trials following different outcomes. **h** Left, population-averaged responses to the no-go stimulus following different outcomes. Right, FA rate of no-go trials following different outcomes. **i** Mean population response to the no-go cue. **j** Correlation of response to the no-go stimulus. **k** Accuracy of decoding stimulus identities from neuronal activity in the T1 and T2 phases in Uncertain sessions using classifiers trained by the population activity of different trial phases. $^{**}P < 10^{-2}$, $^{***}P < 10^{-3}$, $^{\$}P < 0.05$, $^{\$\$\$}P < 10^{-3}$. Data analyzed by (**d**, n = 6 mice) two-sided one-way repeated measures ANOVA with post hoc Tukey's multiple comparisons, (**g,** n = 6 mice) two-sided two-way repeated measures ANOVA with post-hoc Bonferroni comparisons, (**h,** n = 6 mice) two-sided paired t-test, (**i,** n = 594 neurons from 6 mice) two-sided Friedman test with post-hoc Bonferroni comparisons, or (**k,** n = 500 times repeat) two-sided Kruskal-Wallis test with post-hoc Bonferroni comparisons. Data are presented as (**c**, **d**, **e**, **g**, **h**) mean ± s.e.m. or (**f**, **i**, **k**) box plots (center line, median; box limits, upper and lower quartiles; whiskers, 1.5 × interquartile range). Statistical details are presented in Supplementary Table 1. Source data are provided as a Source data file.

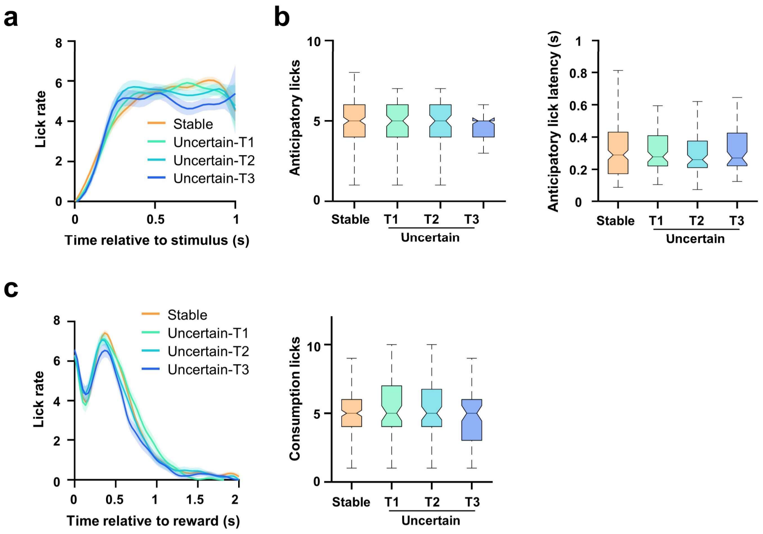

Supplementary Fig. 5 The anticipatory and consumption licking rates are stable throughout the Uncertain session. **a**. The anticipatory licking rate of the Hit trials in the Stable session and T1-T3 phases of the Uncertain session (mean ± s.e.m.). **b** The number of anticipatory licks (left) and lick latency (right) in each phase (n = 67~194 trials from 6 mice. Two-sided Kruskal-Wallis test with post-hoc Bonferroni comparisons). **c** The consumption licking rate relative to the onset of reward delivery in the Stable session and T1-T3 phases of the Uncertain session (left, mean ± s.e.m.), and the number of total consumption licks in each phase (right, n = 70~194 trials from 6 mice. Two-sided Kruskal-Wallis test with post-hoc Bonferroni comparisons). In box plot: center line, median; box limits, upper and lower quartiles; whiskers,1.5 × interquartile range. Statistical details are presented in Supplementary Table 1. Source data are provided as a Source data file.

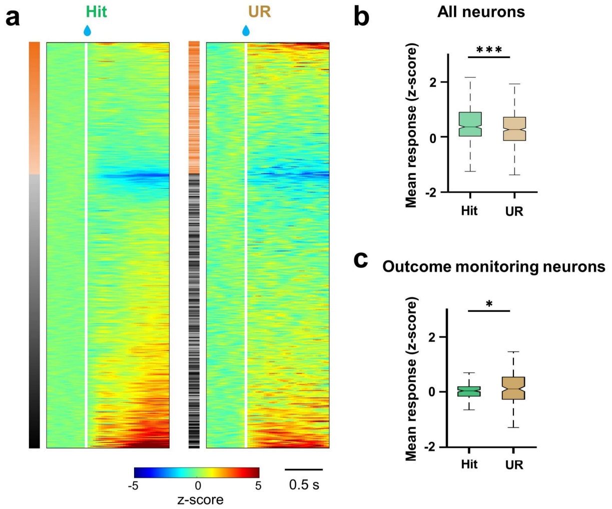

Supplementary Fig. 6 Outcome monitoring neurons detect the unexpected reward. a Trial-averaged neuronal response to reward in the Hit (left) and UR (right) trials. White vertical lines denote the time of reward delivery. Each row represents one neuron and they were sorted by their functional category, indicated by the vertical color bars to the left of the heat map (orange bar, outcome monitoring neurons identified in Fig. 3g; black bar, other neurons). **b** Mean population responses of all recorded neurons during 1-s window after reward delivery in the Hit and UR trials (n = 549 neurons from 6 mice, two-sided Wilcoxon signed-rank test). **c** Mean population responses of identified outcome monitoring neurons (marked by light orange vertical bars adjacent to heat maps in **a**) during 1-s window after reward delivery in the Hit and UR trials (n = 179 neurons from 6 mice, two-sided Wilcoxon signed-rank test). Results suggest that outcome monitoring neurons not only monitored unexpected omission of reward (RO, Fig. 3c) but also the unexpected granting of reward. $*P < 0.05$, $***P < 10^{-3}$. In box plot: center line, median; box limits, upper and lower quartiles; whiskers, 1.5 × interquartile range. Statistical details are presented in Supplementary Table 1. Source data are provided as a Source data file.

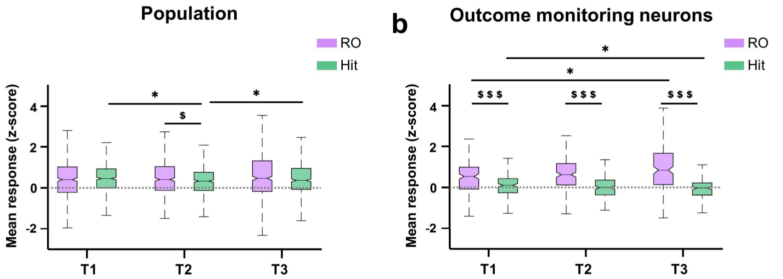

Supplementary Fig. 7 The difference of outcome evoked activity in the Hit and RO trials emerges with time in the Uncertain session. **a** Mean response of all neurons during 1-s window after reward omission or delivery during RO and Hit trials in the T1-T3 phases of the Uncertain session (n = 549 neurons from 6 mice. The symbol * denotes significant differences between the trials following the same outcomes in different phases (two-sided Friedman test with post-hoc Bonferroni comparisons) and the symbol $ denotes significant differences between the trials following different outcomes in the same phase (two-sided Wilcoxon signed-rank test)). **b** Mean responses of outcome monitoring neurons during 1-s window after reward omission or delivery during RO and Hit trials in the T1-T3 phases of the Uncertain session (n = 179 neurons from 6 mice. The symbol * denotes significant differences between the trials following the same outcomes in different phases (two-sided Friedman test with post-hoc Bonferroni comparisons) and the symbol $ denotes significant differences between the trials following different outcomes in the same phase (two-sided Wilcoxon signed-rank test). Note that there were only 1~6 RO trials and 4~12 Hit trials in each phase. $^{*}P < 0.05$, $^{\$}P < 0.05$, $^{\$\$\$}P < 10^{-3}$.   In box plot: center line, median; box limits, upper and lower quartiles; whiskers, 1.5 × interquartile range. Statistical details are presented in Supplementary Table 1. Source data are provided as a Source data file

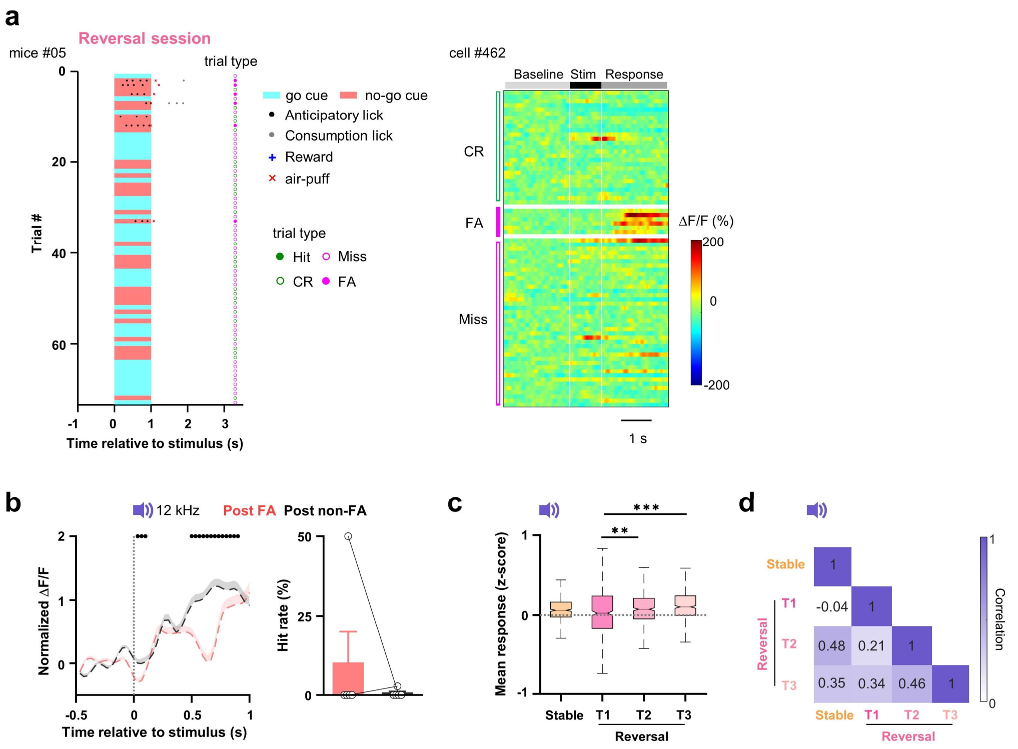

Supplementary Fig. 8 Go cue evoked response in the ACC neurons remains low in the Reversal session. a Licking performance and trial outcomes of an example mouse in the Reversal session (left, the same example mouse in Fig. 4c), and the response of an example neuron (right). Each row represents the response of one trial and all trials are sorted by the trial type. **b** Left, population-averaged go stimulus-evoked responses in the trials following FA trials and following other non-FA trials (red and black traces respectively). Black dots indicate the time segments when two traces are different ($P < 0.05$, two-sided Wilcoxon rank-sum test). Right, the Hit rate of trials following FA and non-FA trials (red and black bars respectively. n = 5 mice, two-sided paired t-test). Noteworthy, ACC responses to go and no-go stimulus both decreased following an FA trial, possibly indicating disassociation of stimulus to the lick decision after being repeatedly punished upon licking. **c** Mean population response to the 12 kHz tone in the Stable session and three phases of the Reversal session (two-sided Friedman test with post-hoc Bonferroni comparisons). **d** Correlation of 12 kHz tone-evoked population activity between the Stable session and T1-T3 phases of the Reversal session. $^{**}P < 10^{-2}$, $^{***}P < 10^{-3}$. Data are presented as (**b**) mean ± s.e.m. or (**c**) box plots (center line, median; box limits, upper and lower quartiles;

whiskers, 1.5 × interquartile range). Statistical details are presented in Supplementary Table 1. Source data are provided as a Source data file.

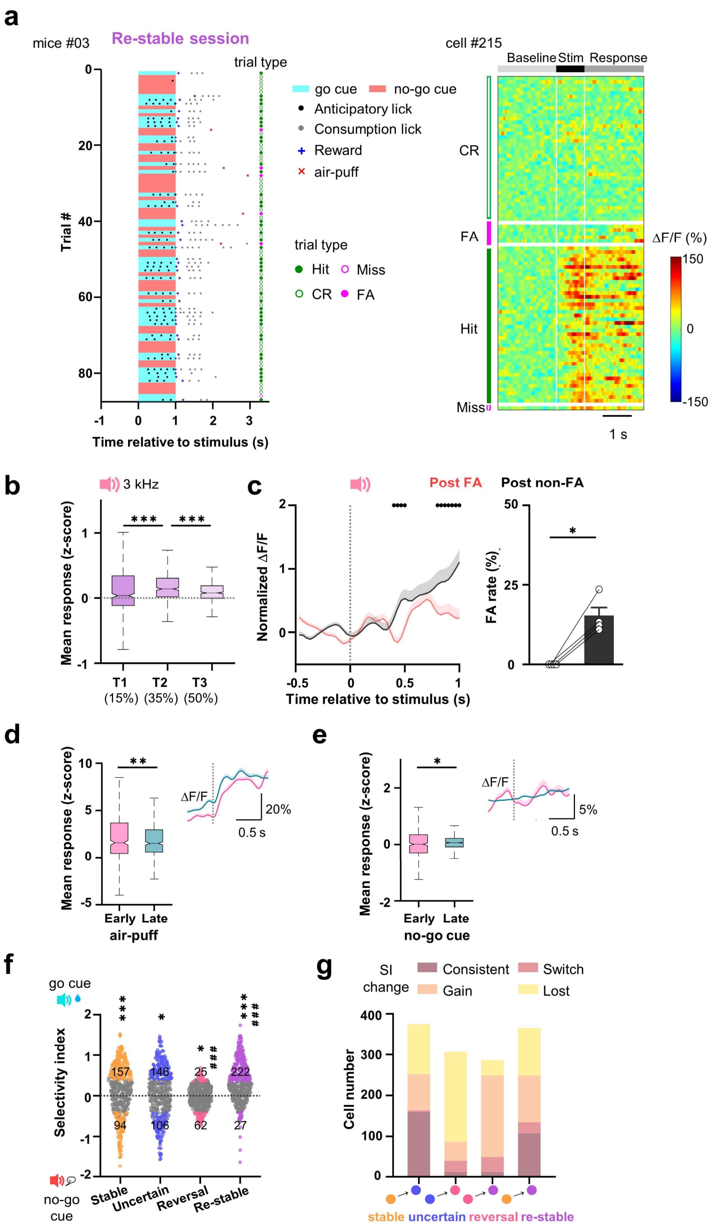

Supplementary Fig. 9 Value representation is consistent throughout the Re-stable session. a Licking performance and trial outcomes of an example mouse in the Re-stable session (left), and the response of an example neuron (right). Each row represents one trial and all trials are sorted by the trial type. **b** Mean population response to no-go stimulus in the Re-stable session (n = 493 neurons from 5 mice, two-sided Friedman test with post-hoc Bonferroni comparisons). **c** Left, the population-averaged no-go stimulus-evoked responses following FA (post FA) and the other (post non-FA) trials. Right, the FA rate of no-go trials following different trials (two-sided paired t-test). **d** The mean normalized

population responses after air-puff delivery in the early (first 15% of trials) and late (last 85% of trials) periods of the Re-stable session (two-sided Wilcoxon signed-rank test). Inset, population-averaged Ca^{2+} traces (mean ± s.e.m.). **e** The mean normalized population responses to the no-go cue in the early and late periods (two-sided Wilcoxon signed-rank test). Inset, population average Ca^{2+} traces (mean ± s.e.m.). **f** Stimulus selectivity index (SI) of individual neurons in four task sessions (n = 493 cells from 5 mice; SI > 0: prefers to response to the go cue, SI < 0: prefers to response to the no-go cue). Symbol *: difference between SI and 0 (two-sided one-sample Wilcoxon signed-rank test), #: difference between the SI of the Stable session and the other sessions (two-sided Kruskal-Wallis test with post-hoc Bonferroni comparisons, comparing with the Stable session). Color dots indices neurons with significant SI in each session ($P < 0.05$, permutation test). **g** The change of stimulus selectivity of longitudinally tracked neurons across sessions. Only neurons with significant selectivity in at least one of the two sessions were included for analysis. *$P < 0.05$, **$P < 10^{-2}$, ***$P < 10^{-3}$, $^{###}P < 10^{-3}$. Data are presented as (**c**) mean ± s.e.m. or (**d**, **e**) box plots (center line, median; box limits, upper and lower quartiles; whiskers, 1.5 × interquartile range). Statistical details are presented in Supplementary Table 1. Source data are provided as a Source data file.

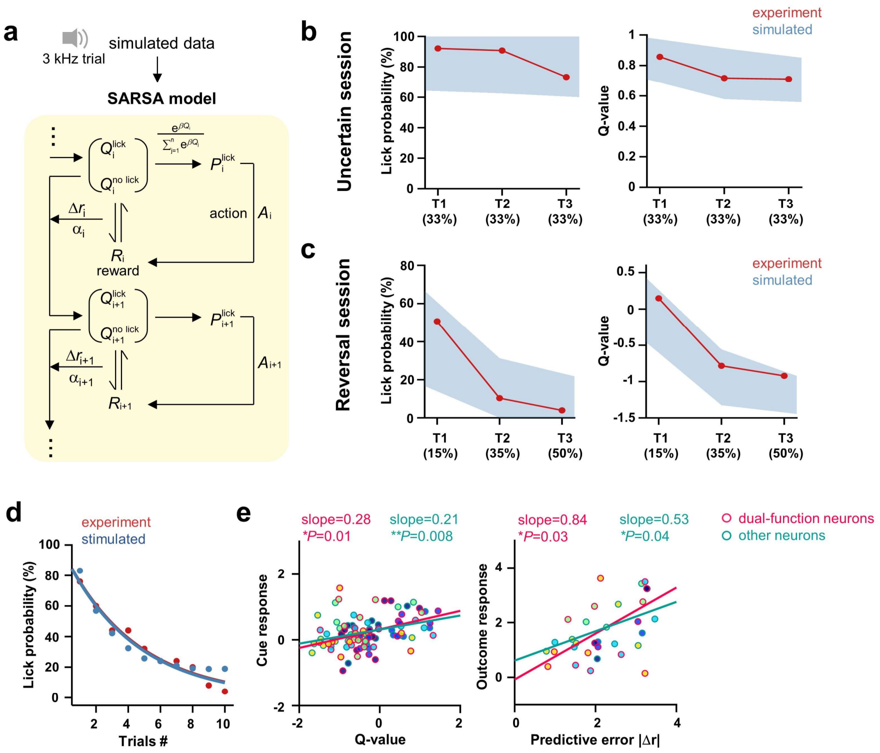

Supplementary Fig. 10 ACC activity in the stimulus and response window correlated well with the Q-value and prediction error estimated by the SARSA model a Schematic diagram of the process of using a SARSA model to simulate trial-by-trial Q-value and action sequence according to the value iteration and QP transformation function. **b** Licking probability and Q-value across T1-T3 phases in the Uncertain session of experiment group and simulated group. Blue shade: 95% distribution of simulated data (3,000 repeats). Mean experiment results (red line) fell within the 95% distribution of the simulated data. (experiment: n = 6 mice, stimulated: n = 3,000 repeats.) **c** Similar to **b**, except for the Reversal session. **d** Licking probability of experiment group and simulated group in the first 10 trials of 3 kHz stimulus in the Reversal session. The lick probability of each animal was calculated by the probability of over 5 adjacent trials. The lick probability of simulated group was calculated by the choice of over 3000 repeats. The decay of the licking probability was fit with an exponential function for each group. **e** Left: relationship between the model estimated Q-value and the mean population responses to 3 kHz stimulus in the first 10 trials in the Reversal session. (n = 5 mice, 10 trials/animal). Right: relationship between the model estimated perdition error (Δr) and the mean

population responses to the air puff in FA trials in the Reversal session (n = 5 mice, 2~5 trials/animal). The activity of dual-function neurons correlated better to the model estimated Q-value and prediction error than the rest of the population, and the slopes of linear correlations were also steeper with the activity of dual-function neurons. Data points from the one mouse were marked with the same color. The data points corresponding to the responses of dual-function neurons and the rest of the population were marked with magenta and cyan outlines, respectively. *P* values test the significance of the correlation. Source data are provided as a Source data file.

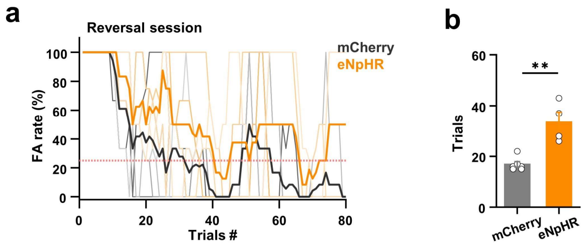

Supplementary Fig. 11 Optogenetic inhibition of ACC delayed decision switch in the Reversal session. a Learning curve (FA rate) in the Reversal session. The thick grey and orange lines represent the mean performance values of mCherry (n = 6 mice) and eNpHR (n = 4 mice) groups, and thin lines represent each animal's performance, respectively. The FA rate was calculated by a sliding window of 5 trials. **b** The number of trials required for each mouse to reach switching threshold (FA < 25%, indicated by the red dashed line in **a**) for the first time (two-sided unpaired t-test). $^{**}P < 10^{-2}$. Data are presented as mean ± s.e.m. Statistical details are presented in Supplementary Table 1. Source data are provided as a Source data file.

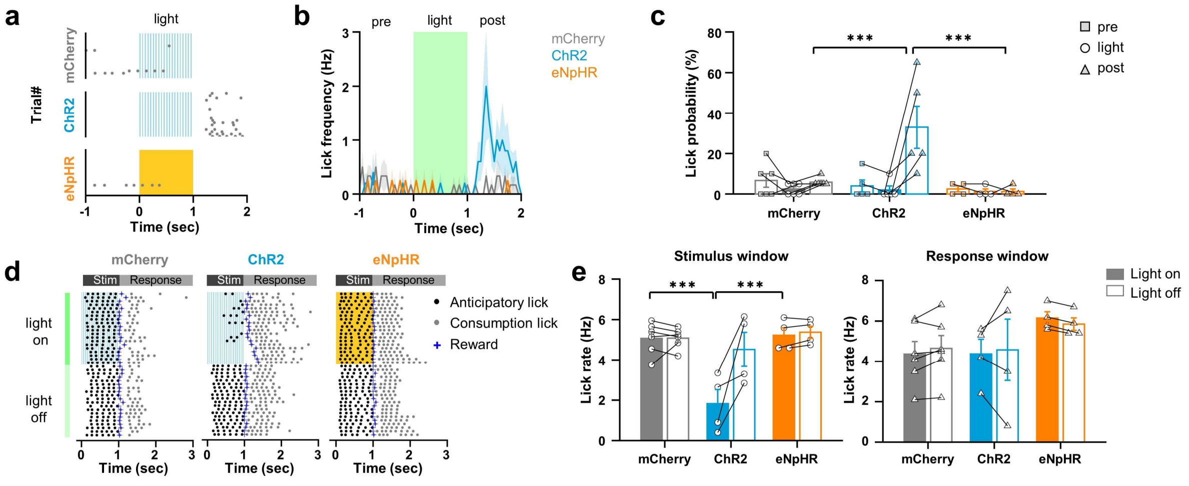

Supplementary Fig. 12. Optogenetic activation of ACC neurons triggers licking response. a. Lick raster of mCherry (top), ChR2 (middle), and eNpHR mice (bottom). blue shade: optogenetic stimulation (488nm, 20 Hz, duration: 1 s); yellow shade: optogenetic stimulation (589 nm, duration: 1 s). **b**. Trial-averaged licking frequency of mCherry, ChR2, and eNpHR mice. Light green shade: optogenetic stimulation. **c** Licking probability of pre-, during and post- optogenetic stimulation. The ChR2 group show higher licking frequency post optogenetic stimulation, illustrating that the photoactivation of ACC triggered rebound licking (mCherry: n = 6 mice; ChR2: n = 5 mice; eNpHR: n = 4 mice. Two-sided two-way repeated measures ANOVA with post hoc Bonferroni multiple comparisons). **d-e** Optogenetic manipulation ACC activity during 3 kHz stimulus presentation in randomly selected 50% of go trials of the Stable session. **d** Raster plots of representative animals from the mCherry (left), ChR2 (middle), and eNpHR group (right) during light on and light off trials. Dark and light gray bars mark the stimulus and response window, respectively. **e** Lick rate during stimulus (left) and response windows (right). (mCherry: n = 6 mice; ChR2: n = 4 mice; eNpHR: n = 4 mice. Two-sided two-way repeated measures ANOVA with post hoc Bonferroni multiple comparisons). Data are presented as mean ± s.e.m. Statistical details are presented in Supplementary Table 1. Source data are provided as a Source data file.

Supplementary Table 1: Statistics table

The table shows the statistics for all main text and supplementary figures. Statistical analyses were performed with scripts written in MATLAB (2020a, MathWorks) and GraphPad Prism 9 (GraphPad).

Figure	Description	Sample size	Statistic	*P* Value	Degrees of freedom & F/t/z/R/ETC Value	mean (s.e.m.) / median (25%, 75%)	effect size & confidence intervals (Cohen d)
**Fig. 1**							
i	*d′*	5 mice	RM one way ANOVA	*P* = 1.3471*10^-05	F (1.6103, 6.4412) = 117.1736	Stable: 3.4380 (0.2096）;<br>Uncertain: 2.6222 (0.0938);<br>Reversal: -0.9195 (0.1297);<br>Re-stable: 2.4016 (0.3247)	
	Stable vs. Uncertain		post hoc Dunnett's multiple comparisons	*P* = 0.0370			1.7927 (0.3204, 4.7789)
	Stable vs. Reversal		post hoc Dunnett's multiple comparisons	*P* < 10^-15			8.9207 (4.1818, 20.574)
	Stable vs. Re-stable		post hoc Dunnett's multiple comparisons	*P* = 0.0509			1.3533 (0.1592, 3.6997)
j	Trajectory length: go cues	5,000 times repeat	Kruskal-Wallis test	*P* = 0	H = 1.3770*10^+04	Stable: 22.5601 (20.1192, 26.7851);<br>Uncertain: 18.6464 (16.5318, 21.6589);<br>Reversal: 11.0652 (10.3185, 11.8614);<br>Re-stable: 26.8537 (22.6590, 43.9956)	
	Stable vs. Uncertain		post hoc Bonferroni multiple comparisons	*P* = 1.4230*10^-183			0.5868 (0.5467, 0.62677)
	Stable vs. Reversal		post hoc Bonferroni multiple comparisons	*P* = 0			2.4509 (2.399, 2.5027)
	Stable vs. Re-stable		post hoc Bonferroni multiple comparisons	*P* = 8.2830*10^-128			-0.6840 (-0.7243, -0.6436)
	Trajectory length: no-go cues	5,000 times repeat	Kruskal-Wallis test	*P* = 0	H = 7.6630*10^+03	Stable: 11.6621 (10.7280, 12.7172);<br>Uncertain: 15.8362 (14.6241, 17.1828);<br>Reversal: 11.6171 (10.6764, 13.4239);<br>Re-stable: 11.7218 (10.3940, 13.3873)	
	Stable vs. Uncertain		post hoc Bonferroni multiple comparisons	*P* = 0			-2.1272 (-2.1762, -2.0781)
	Stable vs. Reversal		post hoc Bonferroni multiple comparisons	*P* = 4.0718*10^-10			-0.2493 (-0.2887, -0.21)
	Stable vs. Re-stable		post hoc Bonferroni multiple comparisons	*P* = 4.2351*10^-06			-0.1869 (-0.226, -0.1476)
	Trajectory length: go cue vs. no-go cue						
	Stable	5,000 times repeat	Wilcoxon rank-sum test (two tailed)	*P* = 0	Z = 86.5610		
	Uncertain	5,000 times repeat	Wilcoxon rank-sum test (two tailed)	*P* = 0	Z = 46.2434		
	Reversal	5,000 times repeat	Wilcoxon rank-sum test (two tailed)	*P* = 8.5293*10^-129	Z = -24.1445		
	Re-stable	5,000 times repeat	Wilcoxon rank-sum test (two tailed)	*P* = 0	Z = 86.2385		
k	Trajectory distance	5,000 times repeat	Kruskal-Wallis test	*P* = 0	H = 1.1915*10^+04	Stable: 136.5987 (112.5600, 180.0130);<br>Uncertain: 122.8232 (98.1380, 160.6658);<br>Reversal: 50.2587 (40.2866, 60.0921);<br>Re-stable: 167.0533 (133.0411, 257.1030)	
	Stable vs. Uncertain		post hoc Bonferroni multiple comparisons	*P* = 1.5937*10^-31			0.2605 (0.2212, 0.30)
	Stable vs. Reversal		post hoc Bonferroni multiple comparisons	*P* = 0			2.1085 (2.0596, 2.1573)
	Stable vs. Re-stable		post hoc Bonferroni multiple comparisons	*P* = 4.2344*10^-69			-0.4964 (-0.5362, -0.4566)
**Fig. 2**							
c	Hit rate change	6 mice	RM one-way ANOVA	*P* = 0.0660	F (3, 15) = 2.9609	Stable: 96.6226 (1.7869);<br>Uncertain T1: 92.1218 (3.1816);<br>Uncertain T2: 90.7407 (6.5210);<br>Uncertain T3: 73.3669 (8.2120)	
	Stable vs. Uncertain		post hoc Dunnett's multiple comparisons	*P* = 0.9087			0.6571 (-0.4401, 1.7242)
	Stable vs. Reversal		post hoc Dunnett's multiple comparisons	*P* = 0.8250			0.4634 (-0.6108, 1.5157)
	Stable vs. Re-stable		post hoc Dunnett's multiple comparisons	*P* = 0.0367			1.4742 (0.2245, 2.6712)
	FA rate change	6 mice	RM one-way ANOVA	*P* = 0.1144	F (3, 15) = 2.3416	Stable: 7.6263 (2.7526);<br>Uncertain T1: 6.2500 (4.2696);<br>Uncertain T2: 11.8519 (4.7633);<br>Uncertain T3: 0 (0)	
	Stable vs. Uncertain T1		post hoc Dunnett's multiple comparisons	*P* = 0.9806			0.1443 (-0.9052, 1.1868)

	Stable vs. Uncertain T2		post hoc Dunnett's multiple comparisons	*P* = 0.6803			-0.4092 (-1.4584, 0.6597)
	Stable vs. Uncertain T3		post hoc Dunnett's multiple comparisons	*P* = 0.2568			1.476 (0.2259, 2.6734)
d	Mean response (z-score) to go cue	549 neurons from 6 mice	Friedman test	*P* = 1.8023*10^-05	H = 24.6787	Stable: 0.2045 (-0.1229, 0.5618); Uncertain T1: 0.1583 (-0.1584, 0.5134); Uncertain T2: 0.1801 (-0.1957, 0.5931); Uncertain T3: 0.0924 (-0.1768, 0.5058)	
	Stable vs. Uncertain T1		post hoc Bonferroni multiple comparisons	*P* = 1			0.0633 (0.0029, 0.1239)
	Stable vs. Uncertain T2		post hoc Bonferroni multiple comparisons	*P* = 0.4312			-0.0057 (-0.0778, 0.0664)
	Stable vs. Uncertain T3		post hoc Bonferroni multiple comparisons	*P* = 0.0113			0.0951 (0.0343, 0.1563)
	Uncertain T1 vs. Uncertain T2		post hoc Bonferroni multiple comparisons	*P* = 0.1239			-0.0703 (-0.1209, -0.0202)
	Uncertain T1 vs. Uncertain T3		post hoc Bonferroni multiple comparisons	*P* = 0.0568			0.0331 (-0.0169, 0.0832)
	Uncertain T2 vs. Uncertain T3		post hoc Bonferroni multiple comparisons	*P* = 5.4923*10^-06			0.1028 (0.0488, 0.1573)
e	Hit rate: post normal outcomes vs. post unexcepted outcomes	6 mice	Paired t test (two tailed)	*P* = 0.5817	t5 = -0.5886	post normal outcomes: 84.9036 (3.7941); post unexcepted outcomes: 88.7554 (4.0866);	-0.368 (-1.4154, 0.6971)
i	Classification accuracy: test for T3 phase	500 times repeat	Kruskal-Wallis test	*P*= 1.0425*10^-28	H = 133.3174	Classifier I: 83.3333 (80.0000, 87.7778); Classifier II: 84.4444 (80.0000, 87.7778); Classifier III: 86.6667 (83.3333, 90.0000); Classifier IV: 87.7778 (83.3333, 91.1111)	
	Classifier I vs. Classifier II		post hoc Bonferroni multiple comparisons	*P* = 0.0403			-0.159 (-0.283, -0.0349)
	Classifier I vs. Classifier III		post hoc Bonferroni multiple comparisons	*P*= 8.6497*10^-16			-0.5375 (-0.6635, -0.4113)
	Classifier I vs. Classifier IV		post hoc Bonferroni multiple comparisons	*P*= 3.1126*10^-23			-0.649 (-0.776, -0.5218)
	Classifier II vs. Classifier III		post hoc Bonferroni multiple comparisons	*P*= 1.7091*10^-07			-0.3688 (-0.4937, -0.2438)
	Classifier II vs. Classifier IV		post hoc Bonferroni multiple comparisons	*P*= 8.4577*10^-13			-0.4804 (-0.6059, -0.3546)
	Classifier III vs. Classifier IV		post hoc Bonferroni multiple comparisons	*P*= 0.3903			-0.1184 (-0.2424, 0.0056)
**Fig. 3**							
b	Mean reward response (z-score) for RO vs. Hit: population	549 neurons from 6 mice	Wilcoxon signed-rank test (two tailed)	*P* = 0.0123	Z = 2.5023	RO: 0.5253 (0.0811, 0.9123); Hit: 0.3550 (0.0240, 0.8847)	0.1097 (0.0324, 0.1876)
c	Mean reward response (z-score) for RO vs. Hit: outcome monitoring neurons	179 neurons from 6 mice	Wilcoxon signed-rank test (two tailed)	*P* = 3.9902*10^-31	Z = 11.6028	RO: 0.6464 (0.4401, 1.0452); Hit: 0.0292 (-0.1623, 0.1861)	1.2756 (1.0726, 1.4978)
e	Mean stimulus response (z-score) for Early vs. Late: population	549 neurons from 6 mice	Wilcoxon signed-rank test (two tailed)	*P*= 2.1783*10^-04	Z = 3.6974	Early: 0.2541 (-0.1747, 0.7041); Late: 0.1855 (-0.2509, 0.7301)	0.0444 (0.0006, 0.0885)
f	Mean stimulus response (z-score) for Early vs. Late: value updating neurons	118 neurons from 6 mice	Wilcoxon signed-rank test (two tailed)	*P* = 4.2090*10^-21	Z = 9.4273	Early: 0.4071 (0.1593, 0.6993); Late: 0.0146 (-0.2131, 0.1729)	0.7166 (0.5536, 0.8962)
h	Classification accuracy: outcome decoding	1,000 times repeat	Wilcoxon rank-sum test (two tailed)	*P* = 1.9137*10^-23	Z= 9.9774	1: 73.6500 (69.8500, 76.9000); 2: 71.2000 (67.5000, 74.7000)	0.4793 (0.3904, 0.5681)
	Classification accuracy: period decoding	1,000 times repeat	Wilcoxon rank-sum test (two tailed)	*P* = 4.4397*10^-18	Z= 8.6669	1: 56.6000 (54.2000, 59.3000); 2: 55.3000 (52.7000, 57.6000)	0.4133 (0.3247, 0.5018)
**Fig. 4**							
c	3 kHz licking (%) change	5 mice	RM one-way ANOVA	*P* = 8.2952*10^-05	F (1.348, 5.894) = 98.61	Stable: 95.9471 (2.0261); Reversal T1: 50.6667 (8.8187); Reversal T2: 10.3173 (2.2186); Reversal T3: 3.9234 (2.4133)	
	Stable vs. Reversal T1		post hoc Dunnett's multiple comparisons	*P* = 0.0061			2.857 (1.0518, 4.596)
	Stable vs. Reversal T2		post hoc Dunnett's multiple comparisons	*P* < 10^-15			16.271 (8.4015, 24.16)
	Stable vs. Reversal T3		post hoc Dunnett's multiple comparisons	*P* < 10^-15			16.673 (8.6135, 24.754)

	12 kHz licking (%) change	5 mice	RM one-way ANOVA	*P* = 0.1281	F (1.4680, 5.8721) = 3.0583	Stable:6.5710 (3.1136);<br>Reversal T1: 0 (0);<br>Reversal T2: 2.7885 (1.7227);<br>Reversal T3: 0 (0)	
	Stable vs. Reversal T1		post hoc Dunnett's multiple comparisons	*P* = 0.2147			1.2049 (-0.0846, 2.4365)
	Stable vs. Reversal T2		post hoc Dunnett's multiple comparisons	*P* = 0.6246			0.6069 (-0.5671, 1.746)
	Stable vs. Reversal T3		post hoc Dunnett's multiple comparisons	*P* = 0.2147			1.2049 (-0.0846, 2.4365)
d	Mean response (z-score) to 3 kHz tone	493 neurons from 5 mice	Friedman test	*P* = 3.4997*10^-31	H = 144.7923	Stable: 0.2123 (-0.1440, 0.5892);<br>Reversal T1: 0.2560 (0.0121, 0.5668);<br>Reversal T2: 0.0241 (-0.0899, 0.1633);<br>Reversal T3: 0.1092 (-0.0511, 0.3077)	
	Stable vs. Reversal T1		post hoc Bonferroni multiple comparisons	*P* = 1.2390*10^-06			-0.0909 (-0.1958, 0.0135)
	Stable vs. Reversal T2		post hoc Bonferroni multiple comparisons	*P* = 5.9206*10^-11			0.2758 (0.1611, 0.3922)
	Stable vs. Reversal T3		post hoc Bonferroni multiple comparisons	*P* = 1			0.1447 (0.0209, 0.2694)
	Reversal T1 vs. Reversal T2		post hoc Bonferroni multiple comparisons	*P* = 2.4424*10^-32			0.5734 (0.4719, 0.6782)
	Reversal T1 vs. Reversal T3		post hoc Bonferroni multiple comparisons	*P* = 4.5275*10^-07			0.3661 (0.262, 0.4724)
	Reversal T2 vs. Reversal T3		post hoc Bonferroni multiple comparisons	*P* = 2.2893*10^-10			-0.2772 (-0.3818, -0.1742)
e	FA rate: post FA vs. post non-FA	5 mice	Paired t test (two tailed)	*P* = 0.4597	t4 = 0.8172	post FA: 16.6667 (6.9722);<br>post non-FA: 11.8555 (2.0678)	0.3338 (-0.9664, 1.9347)
h	Mean punishment response (z-score) for Early vs. Late: population	493 neurons from 5 mice	Wilcoxon signed-rank test (two tailed)	*P* = 2.0632*10^-06	Z = 4.7471	Early: 1.1743 (0.3701, 2.3185);<br>Late: 0.8576 (0.1480, 1.8911)	0.1676 (0.0847, 0.2515)
i	Mean punishment response (z-score) for Early vs. Late: outcome monitoring neurons	167 neurons from 5 mice	Wilcoxon signed-rank test (two tailed)	*P* = 3.7165*10^-29	Z = 11.2082	Early: 1.5537 (0.7612, 2.8585);<br>Late: 0.2866 (-0.2485, 0.6369)	0.9241 (0.7516, 1.1118)
k	Mean stimulus response (z-score) for Early vs. Late: population	493 neurons from 5 mice	Wilcoxon signed-rank test (two tailed)	*P* = 9.6186*10^-24	Z = 10.0455	Early: 0.3832 (-0.0218, 0.7574);<br>Late: 0.0765 (-0.0907, 0.2801)	0.4913 (0.3902, 0.5952)
l	Mean stimulus response (z-score) for Early vs. Late: value updating neurons	281 neurons from 5 mice	Wilcoxon signed-rank test (two tailed)	*P* = 7.8086*10^-48	Z = 14.5301	Early: 0.5785 (0.3585, 0.9851);<br>Late: 0.0307 (-0.1081, 0.1901)	1.2697 (1.1179, 1.4335)
o	Learning rate	5 mice	RM one-way ANOVA	*P* = 1.1540*10^-04	F (1.1191, 4.70463) = 152.1853	Stable: 0.0114 (0.0005);<br>Uncertain: 0.0612 (0.0032);<br>Reversal: 0.1528 (0.0108)	
	Stable vs. Uncertain		post hoc Tukey's multiple comparisons	*P* = 1.6027*10^-04			-8.707 (-13.022, -4.3762)
	Stable vs. Reversal		post hoc Tukey's multiple comparisons	*P* = 3.6770*10^-04			-7.4689 (-11.21, -3.7036)
	Uncertain vs. Reversal		post hoc Tukey's multiple comparisons	*P* = 0.0014			-4.6406 (-7.1098, -2.1228)
**Fig. 5**							
c	Hit rate change: Stable	5 mice	RM one-way ANOVA	*P* = 0.5706	F (2, 8) = 0.6024	T1: 98.3333 (1.6667);<br>T2: 93.9881 (3.0590);<br>T3: 95.5556 (4.4444)	
	T1 vs. T2		post hoc Tukey's multiple comparisons	*P* = 0.5496			0.7121 (-0.4782, 1.8626)
	T1 vs. T3		post hoc Tukey's multiple comparisons	*P* = 0.7742			0.3341 (-0.8064, 1.4546)
	T2 vs. T3		post hoc Tukey's multiple comparisons	*P* = 0.9200			-0.1659 (-1.2826, 0.961)
	FA rate change: Stable	5 mice	RM one-way ANOVA	*P* = 0.6116	F (2, 8) = 0.5232	T1: 10.0346 (6.7613);<br>T2: 4 (4);<br>T3: 4.7348 (1.9882)	
	T1 vs. T2		post hoc Tukey's multiple comparisons	*P* = 0.6335			0.4386 (-0.7132, 1.5643)
	T1 vs. T3		post hoc Tukey's multiple comparisons	*P* = 0.7000			0.4293 (-0.7213, 1.5545)
	T2 vs. T3		post hoc Tukey's multiple comparisons	*P* = 0.9928			-0.0939 (-1.2109, 1.0289)

	Hit rate change: Re-stable	5 mice	RM one-way ANOVA	*P* = 0.0768	F (2, 8) = 3.5995	T1: 75.6432 (9.1442); T2: 85.6692 (6.2214); T3: 89.2457 (3.1758)	
	T1 vs. T2		post hoc Tukey's multiple comparisons	*P* = 0.1984			-0.5175 (-1.6489, 0.644)
	T1 vs. T3		post hoc Tukey's multiple comparisons	*P* = 0.0743			-0.8023 (-1.9642, 0.4035)
	T2 vs. T3		post hoc Tukey's multiple comparisons	*P* = 0.7810			-0.2923 (-1.4113, 0.8443)
	FA rate change: Re-stable	5 mice	RM one way ANOVA	*P* = 0.5944	F (2, 8) = 0.5556	T1: 14.0000 (6.3596); T2: 13.3981 (2.3716); T3: 8.3529 (5.8718)	
	T1 vs. T2		post hoc Tukey's multiple comparisons	*P* = 0.9943			0.0506 (-1.0702, 1.1683)
	T1 vs. T3		post hoc Tukey's multiple comparisons	*P* = 0.6206			0.3725 (-0.7719, 1.4946)
	T2 vs. T3		post hoc Tukey's multiple comparisons	*P* = 0.6802			0.4548 (-0.6988, 1.5817)
d	Mean response (z-score) to go cue: Stable	493 neurons from 5 mice	Friedman test	*P* = 0.1884	H = 3.3387	T1: 0.2268 (-0.2221, 0.7332); T2: 0.2120 (-0.2330, 0.6250); T3: 0.1548 (0.1560, 0.5369)	
	T1 vs. T2		post hoc Bonferroni multiple comparisons	*P* = 0.2932			0.043 (-0.0085, 0.0947)
	T1 vs. T3		post hoc Bonferroni multiple comparisons	*P* = 0.4033			0.061 (-0.0044, 0.1268)
	T2 vs. T3		post hoc Bonferroni multiple comparisons	*P* = 1			0.0154 (-0.0382, 0.0691)
e	Mean response (z-score) to go cue: Re-stable	493 neurons from 5 mice	Friedman test	*P* = 2.1077*10^-09	H = 39.9554	T1: 0.3596 (0.0846, 0.7406); T2: 0.2424 (-0.0604, 0.6810); T3: 0.3571 (0.0958, 0.8431)	
	T1 vs. T2		post hoc Bonferroni multiple comparisons	*P* = 8.5463*10^-06			0.0752 (0.0125, 0.1384)
	T1 vs. T3		post hoc Bonferroni multiple comparisons	*P* = 0.5431			-0.0065 (-0.081, 0.0679)
	T2 vs. T3		post hoc Bonferroni multiple comparisons	*P* = 5.2654*10^-09			-0.0989 (-0.1415, -0.0569)
j	Regression coefficients associated with prior outcome history terms: Stable	493 neurons from 5 mice				1 trial prior: -0.0945 (0.0420); 2 trial prior: 0.0936 (0.0307); 3 trial prior: 0.1053 (0.0290); 4 trial prior: 0.0452 (0.0321); 5 trial prior: -0.1153 (0.0316)	
	1 trial prior		One sample Wilcoxon test	*P* = 0.0047	Z = -2.8247		-0.1012 (-0.1895, -0.0127)
	2 trial prior		One sample Wilcoxon test	*P* = 3.0865*10^-05	Z = 4.1670		0.1372 (0.0486, 0.2257)
	3 trial prior		One sample Wilcoxon test	*P* = 3.4000*10^-05	Z = 4.1449		0.1634 (0.0746, 0.252)
	4 trial prior		One sample Wilcoxon test	*P* = 0.1740	Z = 1.3595		0.0633 (-0.0249, 0.1515)
	5 trial prior		One sample Wilcoxon test	*P* = 1.5552*10^-04	Z = -3.8554		-0.1642 (-0.2529, -0.0754)
	Regression coefficients associated with prior outcome history terms: Re-stable	493 neurons from 5 mice				1 trial prior: 0.4197 (0.0646); 2 trial prior: 0.1081 (0.0774); 3 trial prior: 0.0387 (0.0719); 4 trial prior: 0.1237 (0.0710); 5 trial prior: -0.1421 (0.0846)	
	1 trial prior		One sample Wilcoxon test	*P* = 4.9521*10^-19	Z = 8.9133		0.2922 (0.2021, 0.3821)
	2 trial prior		One sample Wilcoxon test	*P* = 0.0869	Z = 1.7118		0.0628 (-0.0254, 0.151)
	3 trial prior		One sample Wilcoxon test	*P* = 0.0018	Z = 3.1265		0.0242 (-0.0639, 0.1124)
	4 trial prior		One sample Wilcoxon test	*P* = 0.0028	Z = 2.9846		0.0783 (-0.01, 0.1665)
	5 trial prior		One sample Wilcoxon test	*P* = 4.6612*10^-08	Z = -5.4638		-0.0755 (-0.1637, 0.0128)
	Absolute regression coefficients associated with prior outcome history terms: Stable vs. Re-stable	493 neurons from 5 mice					
	1 trial prior		Wilcoxon rank-sum test (two tailed)	*P* = 4.7944*10^-06	Z = -4.5736	Stable: 0.5312 (0.0347); Re-stable: 0.8062 (0.0566)	-0.4248 (-0.5508, -0.2985)
	2 trial prior		Wilcoxon rank-sum test (two tailed)	*P* = 2.2811*10^-04	Z = -3.6857	Stable: 0.4662 (0.0228); Re-stable: 0.7553 (0.0697)	-0.0111 (-0.1358, 0.1137)
	3 trial prior		Wilcoxon rank-sum test (two tailed)	*P* = 0.0500	Z = -1.9601	Stable: 0.4727 (0.0202); Re-stable: 0.6970 (0.0647)	0.0547 (-0.0701, 0.1794)
	4 trial prior		Wilcoxon rank-sum test (two tailed)	*P* = 5.1909*10^-07	Z = -5.0191	Stable: 0.4770 (0.0239); Re-stable: 0.7801 (0.0620)	-0.0641 (-0.1889, 0.0607)
	5 trial prior		Wilcoxon rank-sum test (two tailed)	*P* = 0.0115	Z= -2.5262	Stable: 0.5098 (0.0223); Re-stable: 0.7883 (0.0771)	0.0189 (-0.1059, 0.1436)
**Fig. 6**							

b	*d'*	mCherry: 6 mice; eNpHR: 4 mice	RM two-way ANOVA	Time x Group: *P* = 0.2465; Time: *P* = 0.2465; Group: *P* = 0.6602;	Time x Group: F(1, 8) = 1.563; Time: F(1, 8) = 1.563; Group: F(1, 8) = 0.2083	mCherry Light on: 3.6288 (0.3140); mCherry Light off: 3.5354 (0.3260); eNpHR Light on: 3.3918 (0.1560); eNpHR Light off: 3.3918 (0.1560)	
	mCherry: Light on vs. Light off	6 mice	Paired t test with post hoc Bonferroni multiple comparisons (one tailed)	*P* = 0.1788	t5 = 1.5629		0.1001 (-0.0752, 0.3438)
	eNpHR: Light on vs. Light off	4 mice	Paired t test with post hoc Bonferroni multiple comparisons (one tailed)	-	-		0 (0, 0)
	Light on: mCherry vs. eNpHR	mCherry: 6 mice; eNpHR: 4 mice	Unpaired t test with post hoc Bonferroni multiple comparisons (one tailed)	*P* = 0.5805	t8 = 0.5760		0.3356 (-0.8279, 1.479)
	Light off: mCherry vs. eNpHR	mCherry: 6 mice; eNpHR: 4 mice	Unpaired t test with post hoc Bonferroni multiple comparisons (one tailed)	*P* = 0.7445	t8 = 0.3373		0.1966 (-0.9554, 1.3365)
	Hit rate	mCherry: 6 mice; eNpHR: 4 mice	RM two-way ANOVA	Time x Group: *P* = 0.2415; Time: *P* = 0.2415; Group: *P* = 0.1929;	Time x Group: F(1, 8) = 1.600; Time: F(1, 8) = 1.600; Group: F(1, 8) = 2.021	mCherry Light on: 95.8333 (2.7131); mCherry Light off: 94.1667 (3.0046); eNpHR Light on: 100 (0); eNpHR Light off: 100 (0)	
	mCherry: Light on vs. Light off	6 mice	Paired t test with post hoc Bonferroni multiple comparisons (one tailed)	*P* = 0.1747	t5 = 1.5811		0.1998 (-0.1459, 0.6818)
	eNpHR: Light on vs. Light off	4 mice	Paired t test with post hoc Bonferroni multiple comparisons (one tailed)	-	-		NaN (NaN, NaN)
	Light on: mCherry vs. eNpHR	mCherry: 6 mice; eNpHR: 4 mice	Unpaired t test with post hoc Bonferroni multiple comparisons (one tailed)	*P* = 0.2541	t8 = 1.2286		-0.7159 (-1.8888, 0.4972)
	Light off: mCherry vs. eNpHR	mCherry: 6 mice; eNpHR: 4 mice	Unpaired t test with post hoc Bonferroni multiple comparisons (one tailed)	*P* = 0.1590	t8 = 1.5532		-0.905 (-2.1033, 0.3416)
e	*d'*	mCherry: 6 mice; eNpHR: 4 mice	RM two-way ANOVA	Time x Group: *P* = 0.0255; Time: *P* = 0.0259; Group: *P* = 0.5440;	Time x Group: F(1, 8) = 7.505; Time: F(1, 8) = 7.451; Group: F(1, 8) = 0.4016	mCherry Stable: 4.0827 (0.1359); mCherry Uncertain: 3.2027 (0.1894); eNpHR Stable: 3.7696 (0.2450); eNpHR Uncertain: 3.7712 (0.1300);	
	mCherry: Stable vs. Uncertain	6 mice	Paired t test with post hoc Bonferroni multiple comparisons (one tailed)	*P* = 0.0088	t5 = 4.1551		1.8324 (0.4924, 4.3508)
	eNpHR: Stable vs. Uncertain	4 mice	Paired t test with post hoc Bonferroni multiple comparisons (one tailed)	*P* = 0.9950	t3 = -0.0068		-0.0029 (-1.8825, 1.8729)
	Stable: mCherry vs. eNpHR	mCherry: 6 mice; eNpHR: 4 mice	Unpaired t test with post hoc Bonferroni multiple comparisons (one tailed)	*P* = 0.2586	t8 = 1.2152		0.7081 (-0.5037, 1.8801)
	Uncertain: mCherry vs. eNpHR	mCherry: 6 mice; eNpHR: 4 mice	Unpaired t test with post hoc Bonferroni multiple comparisons (one tailed)	*P* = 0.0587	t8 = -2.2027		-1.2835 (-2.5519, 0.0455)
	3 kHz licking	mCherry: 6 mice; eNpHR: 4 mice	RM two-way ANOVA	Time x Group: *P* = 0.2500; Time: *P* = 0.1563; Group: *P* = 0.1760;	Time x Group: F(1, 8) = 1.539; Time: F(1, 8) = 2.448; Group: F(1, 8) = 2.203	mCherry Stable: 99.5833 (0.4167); mCherry Uncertain: 94.3089 (2.9313); eNpHR Stable: 100 (0); eNpHR Uncertain: 99.3902 (0.6098);	
	mCherry: Stable vs. Uncertain	6 mice	Paired t test with post hoc Bonferroni multiple comparisons (one tailed)	*P* = 0.1373	t5 = 1.7678		0.8647 (-0.4692, 2.7855)
	eNpHR: Stable vs. Uncertain	4 mice	Paired t test with post hoc Bonferroni multiple comparisons (one tailed)	*P* = 0.3910	t3 = 1		0.5117 (NaN, NaN)
	Stable: mCherry vs. eNpHR	mCherry: 6 mice; eNpHR: 4 mice	Unpaired t test with post hoc Bonferroni multiple comparisons (one tailed)	*P* = 0.4468	t8 = 0.8000		-0.4662 (-1.6163, 0.7115)
	Uncertain: mCherry vs. eNpHR	mCherry: 6 mice; eNpHR: 4 mice	Unpaired t test with post hoc Bonferroni multiple comparisons (one tailed)	*P* = 0.2064	t8 = -1.3749		-0.8011 (-1.9847, 0.4264)
h	*d'*	mCherry: 6 mice; eNpHR: 4 mice	RM two-way ANOVA	Time x Group: *P* = 0.5774; Time: *P* = 3.5882*10^-10; Group: *P* = 0.6549;	Time x Group：F(1, 8) = 0.3373; Time：F(1, 8) = 1322; Group：F(1, 8) = 0.2154	mCherry Stable: 3.7629 (0.1755); mCherry Reversal: -1.7259 (0.0912); eNpHR Stable: 3.6086 (0.0723); eNpHR Reversal: -1.7076 (0.1901);	
	mCherry: Stable vs. Reversal	6 mice	Paired t test with post hoc Bonferroni multiple comparisons (one tailed)	*P* = 2.2073*10^-06	t5 = 24.7034		13.468 (6.9655, 27.914)

	eNpHR: Stable vs. Reversal	4 mice	Paired t test with post hoc Bonferroni multiple comparisons (one tailed)	*P* = 3.5020*10^-05	t3 = 39.7548		13.373 (5.4352, 36.154)
	Stable: mCherry vs. eNpHR	mCherry: 6 mice; eNpHR: 4 mice	Unpaired t test with post hoc Bonferroni multiple comparisons (one tailed)	*P* = 0.5156	t8 = 0.6802		0.3964 (-0.7734, 1.5425)
	Reversal: mCherry vs. eNpHR	mCherry: 6 mice; eNpHR: 4 mice	Unpaired t test with post hoc Bonferroni multiple comparisons (one tailed)	*P* = 0.9249	t8 = -0.0973		-0.0567 (-1.1973, 1.0874)
	3 kHz licking	mCherry: 6 mice; eNpHR: 4 mice	RM two-way ANOVA	Time x Group: *P* = 0.0395; Time: *P* = 4.8164*10^-07; Group: *P* = 0.0328;	Time x Group：F(1, 8) = 6.034; Time：F(1, 8) = 212.4; Group：F(1, 8) = 6.637	mCherry Stable: 98.75 (1.25); mCherry Reversal: 33.75 (1.6771); eNpHR Stable: 100 (0); eNpHR Reversal: 53.75 (9.4923);	
	mCherry: Stable vs. Reversal	6 mice	Paired t test (two tailed)	*P* = 2.8486*10^-08	t5 = 58.1378		15.085 (7.8957, 31.134)
	eNpHR: Stable vs. Reversal	4 mice	Paired t test (two tailed)	*P* = 0.0165	t3 = 4.8724		2.493 (NaN, NaN)
	Stable: mCherry vs. eNpHR	mCherry: 6 mice; eNpHR: 4 mice	Unpaired t test (two tailed)	*P* = 0.4468	t8 = -0.8000		-0.4662 (-1.6163, 0.7115)
	Reversal: mCherry vs. eNpHR	mCherry: 6 mice; eNpHR: 4 mice	Unpaired t test (two tailed)	*P* = 0.0333	t8 = -2.5669		-1.4957 (-2.8135, -0.1129)
k	*d′*	mCherry: 4 mice; eNpHR: 4 mice	RM two-way ANOVA	Time x Group: *P* = 0.0052; Time: *P* = 0.0025; Group: *P* = 0.4743;	Time x Group: F(1, 6) = 18.28; Time: F(1, 6) = 24.73; Group: F(1, 6) = 0.5824	mCherry Stable: 3.7312 (0.2887); mCherry Uncertain: 2.9391 (0.3132); eNpHR Stable: 3.6019 (0.0456); eNpHR Uncertain: 3.5422 (0.1548);	
	mCherry: Stable vs. Uncertain	4 mice	Paired t test with post hoc Bonferroni multiple comparisons (one tailed)	*P* = 0.0017	t3 = 10.7095		0.9515 (0.3435, 2.638)
	eNpHR: Stable vs. Uncertain	4 mice	Paired t test with with post hoc Bonferroni multiple comparisons (one tailed)	*P* = 0.7250	t3 = 0.3684		0.1893 (-1.8527, 2.4744)
	Stable: mCherry vs. eNpHR	mCherry: 4 mice; eNpHR: 4 mice	Unpaired t test with post hoc Bonferroni multiple comparisons (one tailed)	*P* = 0.6738	t6 = 0.4422		0.2716 (-0.9524, 1.4739)
	Uncertain: mCherry vs. eNpHR	mCherry: 4 mice; eNpHR: 4 mice	Unpaired t test with post hoc Bonferroni multiple comparisons (one tailed)	*P* = 0.1350	t6 = -1.7263		-1.0603 (-2.3649, 0.3131)
	3 kHz licking	mCherry: 4 mice; eNpHR: 4 mice	RM two-way ANOVA	Time x Group: *P* = 0.7304; Time: *P* = 0.1210; Group: *P* = 0.7304;	Time x Group: F(1, 6) = 0.1304; Time: F(1, 6) = 3.261; Group: F(1, 6) = 0.1304	mCherry Stable: 100.0000 (0); mCherry Uncertain: 98.7500 (1.2500); eNpHR Stable: 100 (0); eNpHR Uncertain: 98.1250 (1.1968);	
	mCherry: Stable vs. Uncertain	4 mice	Paired t test with post hoc Bonferroni multiple comparisons (one tailed)	*P* = 0.3910	t3 = 1		0.5117 (NaN, NaN)
	eNpHR: Stable vs. Uncertain	4 mice	Paired t test with with post hoc Bonferroni multiple comparisons (one tailed)	*P* = 0.2152	t3 = 1.5667		0.8016 (NaN, NaN)
	Stable: mCherry vs. eNpHR	mCherry: 4 mice; eNpHR: 4 mice	Unpaired t test with post hoc Bonferroni multiple comparisons (one tailed)	-	-		NaN (NaN, NaN)
	Uncertain: mCherry vs. eNpHR	mCherry: 4 mice; eNpHR: 4 mice	Unpaired t test with post hoc Bonferroni multiple comparisons (one tailed)	*P* = 0.7304	t6 = 0.3612		0.2218 (-0.9972, 1.423)
n	*d′*	mCherry: 4 mice; eNpHR: 4 mice	RM two-way ANOVA	Time x Group: *P* = 0.8697; Time: *P* = 3.2917*10^-08; Group: *P* = 0.0744	Time x Group：F(1, 6) = 0.0293; Time：F(1, 6) = 1265; Group: F(1, 6) = 4.651	mCherry Stable: 3.8430 (0.2640); mCherry Reversal: -1.3794 (0.0872); eNpHR Stable: 3.5197 (0.1320); eNpHR Reversal: -1.7532 (0.0323);	
	mCherry: Stable vs. Reversal	4 mice	Paired t test with post hoc Bonferroni multiple comparisons (one tailed)	*P* = 2.6370*10^-04	t3 = 20.2389		9.6108 (3.8099, 26.134)
	eNpHR: Stable vs. Reversal	4 mice	Paired t test with post hoc Bonferroni multiple comparisons (one tailed)	*P* = 4.3965*10^-05	t3 = 36.8472		19.846 (8.0547, 53.672)
	Stable: mCherry vs. eNpHR	mCherry: 4 mice; eNpHR: 4 mice	Unpaired t test with post hoc Bonferroni multiple comparisons (one tailed)	*P* = 0.3154	t6 = 1.0953		0.6727 (-0.6121, 1.9079)
	Reversal: mCherry vs. eNpHR	mCherry: 4mice; eNpHR: 4 mice	Unpaired t test with post hoc Bonferroni multiple comparisons (one tailed)	*P* = 0.0070	t6 = 4.0187		2.4683 (0.61, 4.2458)

	3 kHz licking	mCherry: 4 mice; eNpHR: 4 mice	RM two-way ANOVA	Time x Group: *P* = 0.0784; Time: *P* = 7.6000*10^-05; Group: *P* = 0.0784	Time x Group：F(1, 6) = 4.488; Time：F(1, 6) = 90.89; Group：F(1, 6) = 4.488	mCherry Stable: 100.0000 (0); mCherry Reversal: 24.3750 (4.1300); eNpHR Stable: 100 (0); eNpHR Reversal: 51.8750 (12.3058);		
	mCherry: Stable vs. Reversal	4 mice	Paired t test with post hoc Bonferroni multiple comparisons (one tailed)	*P* =3.5538*10^-04	t3 = 18.3109			9.369 (NaN, NaN)
	eNpHR: Stable vs. Reversal	4 mice	Paired t test with post hoc Bonferroni multiple comparisons (one tailed)	*P* = 0.0297	t3 = 3.9108			2.001 (NaN, NaN)
	Stable: mCherry vs. eNpHR	mCherry: 4 mice; eNpHR: 4 mice	Unpaired t test with post hoc Bonferroni multiple comparisons (one tailed)	-	-			NaN (NaN, NaN)
	Reversal: mCherry vs. eNpHR	mCherry: 4mice; eNpHR: 4 mice	Unpaired t test with post hoc Bonferroni multiple comparisons (one tailed)	*P* = 0.0784	t6 =2.1186			-1.3013 (-2.6654, 0.1394)
**Supplementary Figure**								
**Supplementary Fig. 2**								
b	Mean response (z-score): go cues	493 neurons from 5 mice	Friedman test	*P* = 1.2340*10^-26	H = 123.6961	Stable: 0.2123 (-0.1440, 0.5892); Uncertain: 0.1364 (-0.1698, 0.5402); Reversal: 0.0803 (-0.0022, 0.1908); Re-stable: 0.3085 (0.0485, 0.7157)		
	Stable vs. Uncertain		post hoc Bonferroni multiple comparisons	*P* = 1				0.0675 (0.0044, 0.131)
	Stable vs. Reversal		post hoc Bonferroni multiple comparisons	*P* = 0.0576				0.1913 (0.0705, 0.3133)
	Stable vs. Re-stable		post hoc Bonferroni multiple comparisons	*P* = 3.1925*10^-13				-0.255 (-0.3625, -0.149)
	Mean response (z-score): no-go cues	493 neurons from 5 mice	Friedman test	*P* = 6.0534*10^-08	H = 36.4369	Stable: 0.0594 (-0.0249, 0.1615); Uncertain: 0.0667 (-0.0335, 0.1932); Reversal: 0.0953 (-0.0188, 0.2444); Re-stable: 0.0323 (-0.0697, 0.1439)		
	Stable vs. Uncertain		post hoc Bonferroni multiple comparisons	*P* = 1				-0.1014 (-0.2098, 0.0064)
	Stable vs. Reversal		post hoc Bonferroni multiple comparisons	*P* = 0.0028				-0.2063 (-0.3155, -0.0983)
	Stable vs. Re-stable		post hoc Bonferroni multiple comparisons	*P* = 0.0818				0.1352 (0.0167, 0.2544)
	Mean response (z-score): go cue vs. no-go cue							
	Stable	493 neurons from 5 mice	Wilcoxon signed-rank test (two tailed)	*P* = 1.6551*10^-05	Z = 4.3070			0.2417 (0.1242, 0.3606)
	Uncertain	493 neurons from 5 mice	Wilcoxon signed-rank test (two tailed)	*P* = 0.0087	Z = 2.6218			0.1373 (0.0163, 0.2592)
	Reversal	493 neurons from 5 mice	Wilcoxon signed-rank test (two tailed)	*P* = 0.2874	Z = -1.0637			-0.0678 (-0.1756, 0.0396)
	Re-stable	493 neurons from 5 mice	Wilcoxon signed-rank test (two tailed)	*P* = 1.4919*10^-36	Z = 12.6274			0.4786 (0.3548, 0.6051)
c	The distribution of response neurons: go cue	493 neurons from 5 mice	chi-squared test	*P* = 0	chi squared = 563.9608	Stable: activated 40.9736; suppressed 21.9067 Uncertain: activated 36.9168; suppressed 23.3266 Reversal: activated 3.8540; suppressed 0.4057 Re-stable: activated 53.5497; suppressed 7.5051		
	Stable vs. Uncertain		post hoc Bonferroni multiple comparisons	*P* = 1	chi squared = 1.7073			
	Stable vs. Reversal		post hoc Bonferroni multiple comparisons	*P* = 0	chi squared = 381.1924			
	Stable vs. Re-stable		post hoc Bonferroni multiple comparisons	*P* = 1.6394*10^-09	chi squared = 43.2304			
	The distribution of response neurons: no-go cue	493 neurons from 5 mice	chi-squared test	*P* = 0	chi squared = 157.9455	Stable: activated 4.0568; suppressed 2.4341 Uncertain: activated 3.2454; suppressed 1.0142 Reversal: activated 18.8641; suppressed 4.5680 Re-stable: activated 5.4767; suppressed 9.1278		

	Stable vs. Uncertain		post hoc Bonferroni multiple comparisons	*P* = 0.7104	chi squared = 3.4565		
	Stable vs. Reversal		post hoc Bonferroni multiple comparisons	*P* = 1.7106*10-12	chi squared = 56.9607		
	Stable vs. Re-stable		post hoc Bonferroni multiple comparisons	*P* = 6.8093*10-05	chi squared = 21.9619		
	The distribution of response neurons: go cue vs. no-go cue	493 neurons from 5 mice					
	Stable		chi-squared test	*P* = 0	chi squared = 346.0134		
	Uncertain		chi-squared test	*P* = 0	chi squared = 354.0410		
	Reversal		chi-squared test	*P* = 4.4409*10^-16	chi squared = 73.5544		
	Re-stable		chi-squared test	*P* = 0	chi squared = 279.3494		
**Supplementary Fig. 3**							
d	Distribution of onsets time point: neural response vs. anticipatory lick	194 trials from 6 mice	Wilcoxon rank-sum test (two tailed)	*P* = 2.3973*10^-08	Z = -5.5806	neural response: 0.2000 (0.0667, 0.4); anticipatory lick: 0.2890 (0.1730, 0.4300);	-0.2516 (-0.3932, -0.1099)
f	Anticipatory lick latency: early licking trials vs. late licking trials	early/late licking: 49/49 trials from 6 mice	Wilcoxon rank-sum test (two tailed)	*P* = 1.4958*10^-17	Z = -8.5275	early licking: 0.2150 (0.1550, 0.2555); late licking: 0.5720 (0.4140, 0.8060);	-2.3943 (-2.909, -1.8722)
g	Neural response latency: early licking trials vs. late licking trials	549 neurons from 6 mice	Wilcoxon rank-sum test (two tailed)	*P* = 0.4897	Z = -0.6907	early licking: 0.2 (0.0667, 0.4); late licking: 0.2 (0.0667, 0.4333)	-0.0335 (-0.0773, 0.0103)
h	Anticipatory licks number: early licking trials vs. late licking trials	early/late licking: 49/49 trials from 6 mice	Wilcoxon rank-sum test (two tailed)	*P* = 4.0303*10^-12	Z = 6.9361	early licking: 5 (5, 6); late licking: 3 (2, 4)	1.7772 (1.3077, 2.2401)
i	mean population response early licking trials vs. late licking trials	549 neurons from 6 mice	Wilcoxon signed-rank test (two tailed)	*P* = 0.2730	Z = -1.0963	early licking: 0.4579 (-1.6323, 3.3576); late licking: 0.7426 (-1.6307, 4.4899)	-0.0266 (-0.0833, 0.03)
k	Anticipatory lick latency: early licking trials vs. late licking trials	early/late licking: 7/9 trials from 5 mice	Wilcoxon rank-sum test (two tailed)	*P* = 6.9930*10^-04	-	early licking: 0.4160 (0.3310, 0.5473); late licking: 1.0000 (0.8245, 1.0000)	-3.026 (-4.4537, -1.5537)
i	Neural response latency: early licking trials vs. late licking trials	493 neurons from 5 mice	Wilcoxon rank-sum test (two tailed)	*P* = 0.6487	Z = -0.4555	early licking: 0.2333 (0.0667, 0.4667); late licking: 0.2333 (0.0667, 0.4667)	-0.0091 (-0.1169, 0.0986)
m	Anticipatory licks number: early licking trials vs. late licking trials	early/late licking: 7/9 trials from 5 mice	Wilcoxon rank-sum test (two tailed)	*P* = 0.0010	-	early licking: 4 (3.25, 5); late licking: 0 (0, 2.25)	2.4131 (1.1041, 3.677)
n	mean population response early licking trials vs. late licking trials	493 neurons from 5 mice	Wilcoxon signed-rank test (two tailed)	*P* = 0.0976	Z = 1.6568	early licking: 3.1106 (-2.5051, 11.1552); late licking: 1.8832 (-2.6641, 8.6386)	-0.0259 (-0.1262, 0.0742)
p	Relative contribution	549 neurons from 6 mice	Kruskal-Wallis test	*P* = 1.2258*10^-15	H = 72.5298	stimulus: 0.3008 (0, 0.7102); cumulative history: 0.1019 (0, 0.5096); previous outcome: 0 (0, 0.4235); licking rate: 0.0064 (0, 0.0652)	
	stimulus vs. cumulative history		post hoc Bonferroni multiple comparisons	*P* = 0.0129			0.258 (0.1349, 0.381)
	stimulus vs. previous outcome		post hoc Bonferroni multiple comparisons	*P* = 4.3461*10^-09			0.447 (0.3228, 0.571)
	stimulus vs. licking rate		post hoc Bonferroni multiple comparisons	*P* =2.0073*10-14			0.7597 (0.6326, 0.8864)
	cumulative history vs. previous outcome		post hoc Bonferroni multiple comparisons	*P* = 0.0119			0.1906 (0.0677, 0.3133)
	cumulative history vs. licking rate		post hoc Bonferroni multiple comparisons	*P* = 9.0854*10^-06			0.516 (0.3913, 0.6404)
	previous outcome vs. licking rate		post hoc Bonferroni multiple comparisons	*P* = 0.5163			0.3345 (0.211, 0.4578)
**Supplementary Fig. 4**							
d	Hit rate change	6 mice	RM one way ANOVA	*P* = 0.1751	F (2, 10) = 2.0846	post normal outcomes: 84.9036 (3.7941); post RO trials: 83.5714 (5.3133); post UR trials: 95.8333 (4.1667);	
	post normal outcomes vs. post RO trials		post hoc Tukey's multiple comparisons	*P* = 0.9778			0.1087 (-0.9392, 1.1513)

	post normal outcomes vs. post UR trials		post hoc Tukey's multiple comparisons	*P* = 0.2675			-1.0333 (-2.1476, 0.124)
	post UR trials vs. post RO trials		post hoc Tukey's multiple comparisons	*P* = 0.2000			-0.9675 (-2.0718, 0.1779)
f	Mean population response of go cue: post normal outcome	549 neurons from 6 mice	Friedman test	*P* = 0.0029	H = 11.6976	post normal outcome-T1: 0.1823 (-0.1482, 0.5388);<br>post normal outcome-T2 0.1822 (-0.2047, 0.6283);<br>post normal outcome-T3: 0.1032 (-0.1626, 0.4841)	
	T1 vs. T2		post hoc Bonferroni multiple comparisons	*P* = 0.4176			0.0613 (-0.1195, -0.0035)
	T1 vs. T3		post hoc Bonferroni multiple comparisons	*P* = 0.1603			0.0489 (-0.0079, 0.1058)
	T2 vs. T3		post hoc Bonferroni multiple comparisons	*P* = 0.0019			0.1112 (0.053, 0.1701)
	Mean population response of go cue: post unexpected outcome	549 neurons from 6 mice	Friedman test	*P* = 3.4123*10^-07	H = 29.7814	post unexpected outcome-T1: 0.1928 (-0.2867, 0.6200);<br>post unexpected outcome-T2: 0.1684 (-0.2529, 0.6782);<br>post unexpected outcome-T3: 0.0424 (-0.4377, 0.5789)	
	post unexpected outcome: T1 vs. T2		post hoc Bonferroni multiple comparisons	*P* = 0.0708			-0.1046 (-0.1749, -0.0348)
	post unexpected outcome: T1 vs. T3		post hoc Bonferroni multiple comparisons	*P* = 0.0046			0.0446 (-0.0349, 0.1244)
	post unexpected outcome: T2 vs. T3		post hoc Bonferroni multiple comparisons	*P* = 1.6704*10^-07			0.1425 (0.0632, 0.2226)
	Mean population response of go cue:post normal outcome vs. post unexpected outcome						
	T1		Wilcoxon signed-rank test (two tailed)	*P* = 0.0231	Z = 2.2715		0.075 (0.007, 0.1434)
	T2		Wilcoxon signed-rank test (two tailed)	*P* = 0.4186	Z = 0.8088		0.0129 (-0.0604, 0.0862)
	T3		Wilcoxon signed-rank test (two tailed)	*P* =5.5068*10^-04	Z = 3.4548		0.0832 (0.018, 0.1488)
g	Hit rate: post normal outcomes vs. post unexcepted outcomes	6 mice	RM two-way ANOVA	Time x Group: *P* = 0.8592;<br>Time: *P* = 0.0648;<br>Group: *P* = 0.5420	Time x Group: F (2, 20) = 0.1529;<br>Time: F (2, 20) = 3.1470;<br>Group: F (1, 10) = 0.3986	post normal outcome-T1: 93.0952 (3.5074);<br>post normal outcome-T2: 90.5556 (7.6214);<br>post normal outcome-T3: 72.1688 (8.3335);<br>post unexcepted outcome-T1: 94.4444 (5.5556);<br>post unexcepted outcome-T2: 91.6667 (8.3333);<br>post unexcepted outcome-T3: 80.5556 (9.0438);	
	T1 vs. T2		post hoc Bonferroni multiple comparisons	*P* = 0.9329			-0.1094 (-1.1519, 0.9385)
	T1 vs. T3		post hoc Bonferroni multiple comparisons	*P* = 0.0747			-0.0524 (-1.0955, 0.9933)
	T2 vs. T3		post hoc Bonferroni multiple comparisons	*P* = 0.1449			-0.3633 (-1.4105, 0.7014)
h	FA rate: post normal outcomes vs. post unexpected outcomes	6 mice	Paired t test (two tailed)	*P* = 0.8513	t5 = 0.1974	post normal outcomes: 6.8205 (2.4781);<br>post unexcepted outcomes: 5.5556 (5.5556);	0.1009 (-1.429, 1.7008)
i	Mean response (z-score) to no-go cue	549 neurons from 6 mice	Friedman test	*P*= 2.5342*10^-18	H = 85.0481	Stable: 0.0490 (0.0349, 0.1474);<br>Uncertain T1: 0.1338 (-0.0429, 0.3100);<br>Uncertain T2: 0.0630 (-0.1096, 0.2555);<br>Uncertain T3: -0.0259 (-0.1929, 0.1776)	
	Stable vs. Uncertain T1		post hoc Bonferroni multiple comparisons	*P*= 8.8098*10^-06			-0.3204 (-0.4321, -0.2103)
	Stable vs. Uncertain T2		post hoc Bonferroni multiple comparisons	*P* = 1			-0.0848 (-0.1956, 0.0257)
	Stable vs. Uncertain T3		post hoc Bonferroni multiple comparisons	*P*= 6.6546*10^-05			0.2073 (0.0987, 0.317)
	Uncertain T1 vs. Uncertain T2		post hoc Bonferroni multiple comparisons	*P*= 8.2439*10^-05			0.1455 (0.035, 0.2568)
	Uncertain T1 vs. Uncertain T3		post hoc Bonferroni multiple comparisons	*P*= 1.9525*10^-19			0.4275 (0.3137, 0.5436)
	Uncertain T2 vs. Uncertain T3		post hoc Bonferroni multiple comparisons	*P*= 6.9633*10^-06			0.2095 (0.1025, 0.3177)

k	Classification accuracy: test for T1 phase	500 times repeat	Kruskal-Wallis test	*P*= 6.4238*10^-37	H = 171.3751	Classifier I: 86.6667 (82.2222, 90.0000); Classifier II: 91.1111 (86.6667, 94.4444); Classifier III: 88.8889 (84.4444 ,92.2222); Classifier IV: 87.7778 (84.4444, 91.1111)	
	Classifier I vs Classifier II		post hoc Bonferroni multiple comparisons	*P*= 7.3033*10^-31			-0.8402 (-0.9692, -0.7108)
	Classifier I vs Classifier III		post hoc Bonferroni multiple comparisons	*P*= 3.8166*10^-14			-0.5067 (-0.6324, -0.3807)
	Classifier I vs Classifier IV		post hoc Bonferroni multiple comparisons	*P*= 1.4551*10^-06			-0.3804 (-0.5053, -0.2553)
	Classifier II vs Classifier III		post hoc Bonferroni multiple comparisons	*P*= 2.9966*10^-06			0.3414 (0.2165, 0.4661)
	Classifier II vs Classifier IV		post hoc Bonferroni multiple comparisons	*P*= 1.1181*10^-13			0.5041 (0.3781, 0.6298)
	Classifier III vs Classifier IV		post hoc Bonferroni multiple comparisons	*P*= 0.0508			0.1478 (0.0238, 0.2718)
	Classification accuracy: test for T2 phase	500 times repeat	Kruskal-Wallis test	*P*= 3.4367*10^-157	H = 726.6823	Classifier I: 86.6667 (83.3333, 91.1111); Classifier II: 91.1111 (87.7778, 94.4444); Classifier III: 96.6667 (94.4444, 97.7778); Classifier IV: 91.1111 (88.8889, 94.4444)	
	Classifier I vs Classifier II		post hoc Bonferroni multiple comparisons	*P*= 1.2495*10^-24			-0.7863 (-0.9147, -0.6576)
	Classifier I vs Classifier III		post hoc Bonferroni multiple comparisons	*P*= 1.9573*10^-156			-1.9089 (-2.0581, -1.759)
	Classifier I vs Classifier IV		post hoc Bonferroni multiple comparisons	*P*=3.7181*10^-29			-0.941 (-1.0714, -0.8102)
	Classifier II vs Classifier III		post hoc Bonferroni multiple comparisons	*P*= 6.2088*10^-59			-1.2317 (-1.3666, -1.0963)
	Classifier II vs Classifier IV		post hoc Bonferroni multiple comparisons	*P*= 1			-0.1215 (-0.2455, 0.0025)
	Classifier III vs Classifier IV		post hoc Bonferroni multiple comparisons	*P*= 2.1913*10^-52			1.2453 (1.1097, 1.3804)
**Supplementary Fig. 5**							
b	Anticipitory licks	Stable/Uncertain-T1/T2/T3: 194/88/79/67 trials from 6 mice	Kruskal-Wallis test	*P* = 0.3485	H = 3.2941	Stable: 5 (4, 6); Uncertain T1: 5 (4, 6); Uncertain T2: 5 (4, 6); Uncertain T3: 5 (4, 5)	
	Stable vs. Uncertain T1		post hoc Bonferroni multiple comparisons	*P* = 1			0.0523 (-0.199, 0.3035)
	Stable vs. Uncertain T2		post hoc Bonferroni multiple comparisons	*P* = 1			0.001 (-0.2598, 0.2619)
	Stable vs. Uncertain T3		post hoc Bonferroni multiple comparisons	*P* = 0.5831			0.2307 (-0.0472, 0.5081)
	Uncertain T1 vs. Uncertain T2		post hoc Bonferroni multiple comparisons	*P* = 1			-0.0506 (-0.353, 0.2519)
	Uncertain T1 vs. Uncertain T3		post hoc Bonferroni multiple comparisons	*P* = 1			0.1714 (-0.1457, 0.4879)
	Uncertain T2 vs. Uncertain T3		post hoc Bonferroni multiple comparisons	*P* = 0.6497			0.2227 (-0.1025, 0.5472)
	Anticipitory lick latency	Stable/Uncertain-T1/T2/T3: 194/88/79/67 trials from 6 mice	Kruskal-Wallis test	*P* = 0.9005	H = 0.5820	Stable: 0.2890 (0.1730, 0.4300); Uncertain T1: 0.2775 (0.2210, 0.4085); Uncertain T2: 0.2600 (0.2107, 0.3753); Uncertain T3: 0.2700 (0.2215, 0.4245)	
	Stable vs. Uncertain T1		post hoc Bonferroni multiple comparisons	*P* = 1			
	Stable vs. Uncertain T2		post hoc Bonferroni multiple comparisons	*P* = 1			
	Stable vs. Uncertain T3		post hoc Bonferroni multiple comparisons	*P* = 1			
	Uncertain T1 vs. Uncertain T2		post hoc Bonferroni multiple comparisons	*P* = 1			
	Uncertain T1 vs. Uncertain T3		post hoc Bonferroni multiple comparisons	*P* = 1			
	Uncertain T2 vs. Uncertain T3		post hoc Bonferroni multiple comparisons	*P* = 1			
c	Consumption licks	Stable/Uncertain-T1/T2/T3: 194/73/79/70 trials from 6 mice	Kruskal-Wallis test	*P* = 0.2602	H = 4.0114	Stable: 5 (4, 6); Uncertain T1: 5 (4, 7); Uncertain T2: 5 (4, 6.75); Uncertain T3: 5 (3, 6)	
	Stable vs. Uncertain T1		post hoc Bonferroni multiple comparisons	*P* = 1			-0.0269 (-0.2781, 0.2244)

	Stable vs. Uncertain T2		post hoc Bonferroni multiple comparisons	*P* = 1			0.1007 (-0.1604, 0.3616)
	Stable vs. Uncertain T3		post hoc Bonferroni multiple comparisons	*P* = 0.5941			-0.1072 (-0.3842, 0.17)
	Uncertain T1 vs. Uncertain T2		post hoc Bonferroni multiple comparisons	*P* = 1			0.13 (-0.1729, 0.4325)
	Uncertain T1 vs. Uncertain T3		post hoc Bonferroni multiple comparisons	*P* = 0.3347			-0.0789 (-0.3951, 0.2376)
	Uncertain T2 vs. Uncertain T3		post hoc Bonferroni multiple comparisons	*P* = 1			-0.2045 (-0.5288, 0.1206)
**Supplementary Fig. 6**							
b	Mean reward response (z-score) for UR vs. Hit: population	549 neurons from 6 mice	Wilcoxon signed-rank test (two tailed)	*P*= 8.9733*10^-04	Z = -3.3209	UR: 0.2587 (-0.1357, 0.7057); Hit: 0.3550 (0.0240,0.8847)	0.1509 (0.0503, 0.2523)
c	Mean reward response (z-score) for UR vs. Hit: outcome monitoring neurons	179 neurons from 6 mice	Wilcoxon signed-rank test (two tailed)	*P*= 0.0202	Z = 2.3220	UR: 0.1042 (-0.2703, 0.5308); Hit: 0.0292 (-0.1623, 0.1861)	-0.2725 (-0.4685, -0.0808)
**Supplementary Fig. 7**							
a	Mean reward response in Hit and RO trials: population: RO	549 neurons from 6 mice	Friedman test	*P* = 0.2201	H = 3.0273	RO-T1: 0.4011 (-0.2203, 1.0169); RO-T2: 0.4066 (-0.1236, 1.0354); RO-T3: 0.4655 (-0.1706, 1.3137);	
	T1 vs. T2			*P* = 1			-0.0388 (-0.1372, 0.0594)
	T1 vs. T3			*P* = 0.2562			-0.112 (-0.2201, -0.0044)
	T2 vs. T3			*P* = 0.8319			-0.08 (-0.191, 0.0306)
	Mean reward response in Hit and RO trials: population: Hit	549 neurons from 6 mice	Friedman test	*P* = 0.0044	H = 10.8670	Hit-T1: 0.4549 (-0.0011, 0.9257); Hit-T2: 0.3294 (-0.1271, 0.7571); Hit-T3: 0.3647 (-0.0717, 0.9504);	
	T1 vs. T2			*P* = 0.0103			0.094 (0.0237, 0.1648)
	T1 vs. T3			*P* = 1			0.0181 (-0.0535, 0.0897)
	T2 vs. T3			*P* = 0.0165			-0.0715 (-0.139, -0.0044)
	Mean reward response in Hit and RO trials: population: RO vs. Hit	549 neurons from 6 mice					
	T1		Wilcoxon signed-rank test (two tailed)	*P* = 0.5215	Z = -0.6410		-0.0153 (-0.1053, 0.0747)
	T2		Wilcoxon signed-rank test (two tailed)	*P* = 0.0195	Z = 2.3358		0.1113 (0.0251, 0.1982)
	T3		Wilcoxon signed-rank test (two tailed)	*P* = 0.1227	Z = 1.5234		0.1221 (0.0084, 0.2365)
b	Mean reward response in Hit and RO trials: outcome monitoring neurons: RO	179 neurons from 6 mice	Friedman test	*P* = 0.0119	H = 8.8603	RO-T1: 0.5340 (-0.0801, 0.9863); RO-T2: 0.6204 (0.1223, 1.1602); RO-T3: 0.8430 (0.1405, 1.6620);	
	T1 vs. T2			*P* = 1			-0.0994 (-0.2907, 0.0902)
	T1 vs. T3			*P* = 0.0110			-0.314 (-0.5183, -0.1148)
	T2 vs. T3			*P* = 0.1338			-0.26 (-0.486, -0.0381)
	Mean reward response in Hit and RO trials: outcome monitoring neurons: Hit	179 neurons from 6 mice	Friedman test	*P* = 0.0305	H = 6.9832	Hit-T1: 0.1044 (-0.2580, 0.4279); Hit-T2: -0.0074 (-0.3627, 0.3575); Hit-T3: -0.0306 (-0.3720, 0.2177);	
	T1 vs. T2			*P* = 0.5592			0.141 (-0.0533, 0.3375)
	T1 vs. T3			*P* = 0.0247			0.265 (0.0874, 0.4469)
	T2 vs. T3			*P* = 0.5592			0.1363 (-0.053, 0.3279)
	Mean reward response in Hit and RO trials: outcome monitoring neurons: RO vs. Hit	179 neurons from 6 mice					
	T1		Wilcoxon signed-rank test (two tailed)	*P* = 1.1400*10^-08	Z = 5.7085		0.4644 (0.2838, 0.6523)
	T2		Wilcoxon signed-rank test (two tailed)	*P* = 1.5729*10^-17	Z = 8.5217		0.8595 (0.6606, 1.0717)
	T3		Wilcoxon signed-rank test (two tailed)	*P* = 2.0938*10^-18	Z = 8.7521		0.9656 (0.7357, 1.2106)
**Supplementary Fig. 8**							
b	Hit rate change: post FA vs. post non-FA	5 mice	Paired t test (two tailed)	*P* = 0.4049	t4 = 0.9302	post FA: 10.0000 (10.0000); post non-FA: 0.5556 (0.5556)	0.4759 (-1.1371, 2.5167)

c	Mean response (z-score) to 12 kHz tone	493 neurons from 5 mice	Friedman test	*P* = 4.4581*10^-05	H = 22.7939	Stable:0.0594 (-0.0249, 0.1615); Reversal T1: 0.0234 (-0.1731, 0.2381); Reversal T2: 0.0723 (-0.0487, 0.2101); Reversal T3: 0.1014 (-0.0034, 0.2397)	
	Stable vs. Reversal T1		post hoc Bonferroni multiple comparisons	*P* = 0.1307			-0.0513 (-0.1768, 0.0739)
	Stable vs. Reversal T2		post hoc Bonferroni multiple comparisons	*P* = 1			-0.137 (-0.2469, -0.028)
	Stable vs. Reversal T3		post hoc Bonferroni multiple comparisons	*P* = 0.1073			-0.1992 (-0.3157, -0.0838)
	Reversal T1 vs. Reversal T2		post hoc Bonferroni multiple comparisons	*P* = 0.0095			-0.0366 (-0.1563, 0.0828)
	Reversal T1 vs. Reversal T3		post hoc Bonferroni multiple comparisons	*P* = 1.8763*10^-05			-0.0605 (-0.1716, 0.0503)
	Reversal T2 vs. Reversal T3		post hoc Bonferroni multiple comparisons	*P* = 0.7943			-0.032 (-0.1354, 0.0713)
**Supplementary Fig. 9**							
b	Mean response (z-score) to 3 kHz tone	493 neurons from 5 mice	Friedman test	*P* = 5.0455*10^-11	H = 47.4199	T1: 0.0362 (-0.1156, 0.3414); T2: 0.1391 (0.0180, 0.3044); T3: 0.0780 (-0.0076, 0.1883)	
	T1 vs. T2		post hoc Bonferroni multiple comparisons	*P* = 3.0422*10^-10			-0.0424 (-0.1309, 0.0457)
	T1 vs. T3		post hoc Bonferroni multiple comparisons	*P* = 0.7160			0.0981 (0.0033, 0.1935)
	T2 vs. T3		post hoc Bonferroni multiple comparisons	*P* = 3.7400*10^-07			0.1889 (0.1125, 0.2663)
c	FA rate change: post FA vs. post non-FA	4 mice	Paired t test (two tailed)	*P* = 0.0143	t3 = -5.1303	post FA: 0 (0); post non-FA: 14.9233 (2.9089)	-2.625 (NaN, NaN)
d	Mean punishment response (z-score) for Early vs. Late: population	422 neurons from 4 mice	Wilcoxon signed-rank test (two tailed)	*P* = 0.0012	Z = 3.2308	Early: 1.5868 (0.4435, 3.6871); Late: 1.5224 (0.5988,2.9566)	0.1776 (0.1004, 0.256)
e	Mean stimulus response (z-score) for Early vs. Late: population	493 neurons from 5 mice	Wilcoxon signed-rank test (two tailed)	*P* = 0.0363	Z = -2.0935	Early: 0.0097 (-0.2991, 0.3483); Late: 0.0652 (-0.0845, 0.2162)	0.0362 (-0.0758, 0.1484)
f	SI					Stable: 0.1570 (-0.2773, 0.5277); Uncertain: 0.0891 (-0.3700, 0.4888); Reversal: -0.0276 (-0.2398, 0.1944); Re-stable: 0.2820 (0.0231, 0.6392)	
	compared with 0						
	Stable		One sample Wilcoxon test	*P* = 3.1251*10^-05	Z = 4.1641		0.1597 (0.0709, 0.2483)
	Uncertain		One sample Wilcoxon test	*P* = 0.0245	Z = 2.2487		0.0905 (0.0021, 0.1787)
	Reversal		One sample Wilcoxon test	*P* = 0.0250	Z = -2.2411		-0.1245 (-0.2129, -0.0359)
	Re-stable		One sample Wilcoxon test	*P* = 4.6958*10^-40	Z = 13.2470		0.6991 (0.6005, 0.7972)
	SI: compared with stable	493 neurons from 5 mice	Kruskal-Wallis test	*P* = 4.4511*10^-32	H = 148.9444		
	Stable vs. Uncertain		post hoc Bonferroni multiple comparisons	*P* = 0.7358			0.0693 (0.0036, 0.1353)
	Stable vs. Reversal		post hoc Bonferroni multiple comparisons	*P* = 2.5391*10^-08			0.2795 (0.1482, 0.4125)
	Stable vs. Re-stable		post hoc Bonferroni multiple comparisons	*P* = 4.3113*10^-09			0.191 (0.059, 0.3242)
**Supplementary Fig. 11**							
b	Number of trials required: mCherry vs. eNpHR	mCherry: 6 mice; eNpHR: 4 mice	Unpaired t test (two tailed)	*P* = 0.0012	t8 = -4.8813	mCherry: 16.8333 (1.0775); eNpHR: 33.5 (3.9686)	-1.943 (-6.4133, 0.1481)
**Supplementary Fig. 12**							
c	Lick probability (%)	mCherry: 6 mice; ChR2: 5 mice; eNpHR: 4 mice	RM two-way ANOVA	Time x Group: *P* = 0.0016; Time: *P* = 0.0052; Group: *P* = 0.0159;	Time x Group: F(4, 24) = 6.074; Time: F(2, 24) = 6.690; Group: F(2, 12) = 5.965	mCherry pre: 6.6667 (3.3333); mCherrylight: 2.5000 (1.1180); mCherry post: 5.8333 (0.8333); ChR2 pre: 4.0000 (2.9155); ChR2 light: 2.0000 (2.0000); ChR2 post: 33 (10.4403); eNpHR pre: 2.5000 (1.4434); eNpHR light: 1.2500 (1.2500); eNpHR post: 1.2500 (1.2500);	
	pre: mCherry vs. ChR2:		post hoc Bonferroni multiple comparisons	*P* = 1			0.4255 (-1.5565, 2.7912)

	pre: mCherry vs.eNpHR:		post hoc Bonferroni multiple comparisons	$P = 1$			0.8862 (-0.4909, 3.3734)
	pre: eNpHR vs. ChR2:		post hoc Bonferroni multiple comparisons	$P = 1$			0.335 (-2.0267, 3.1265)
	light: mCherry vs. ChR2:		post hoc Bonferroni multiple comparisons	$P = 1$			0 (-2.0505, 2.0505)
	light: mCherry vs.eNpHR:		post hoc Bonferroni multiple comparisons	$P = 1$			0.335 (-0.967, 2.0653)
	light: eNpHR vs. ChR2:		post hoc Bonferroni multiple comparisons	$P = 1$			0.2288 (-2.1686, 2.9202)
	post: mCherry vs. ChR2:		post hoc Bonferroni multiple comparisons	$P = 4.3402*10^{-05}$			-1.2991 (-4.0327, 0.299)
	post: mCherry vs.eNpHR:		post hoc Bonferroni multiple comparisons	$P = 1$			1.4472 (-0.6837, 5.3862)
	post: eNpHR vs. ChR2:		post hoc Bonferroni multiple comparisons	$P = 1.8656*10^{-05}$			1.3915 (-0.5277, 5.0435)
e	Lick rate (Hz): stimulus	mCherry: 6 mice; ChR2: 4 mice; eNpHR: 4 mice	RM two-way ANOVA	Time x Group: $P = 0.0032$; Time: $P = 0.0047$; Group: $P = 0.0081$;	Time x Group: $F(2, 11) = 10.13$; Time: $F(1, 11) = 12.49$; Group: $F(2, 11) = 7.713$	mCherry Light on: 5.0750 (0.3286); mCherry Light off: 5.0833 (0.2072); ChR2 Light on: 1.8250 (0.7007); ChR2 Light off: 4.5250 (0.8452); eNpHR Light on: 5.2250 (0.3750); eNpHR Light off: 5.3750 (0.2976)	
	light on: mCherry vs. ChR2:		post hoc Bonferroni multiple comparisons	$P = 1.3517*10^{-04}$			1.8631 (0.6729, 5.1651)
	light on: mCherry vs. eNpHR:		post hoc Bonferroni multiple comparisons	$P = 1$			-0.2028 (-2.4295, 1.7634)
	light on: eNpHR vs. ChR2:		post hoc Bonferroni multiple comparisons	$P = 2.3485*10^{-04}$			-2.1889 (-6.9229, -0.1047)
	light off: mCherry vs. ChR2:		post hoc Bonferroni multiple comparisons	$P = 1$			0.2829 (-2.0052, 2.9342)
	light off: mCherry vs. eNpHR:		post hoc Bonferroni multiple comparisons	$P = 1$			-0.4086 (-3.245, 1.9038)
	light off: eNpHR vs. ChR2:		post hoc Bonferroni multiple comparisons	$P = 0.7183$			-0.4854 (-2.9215, 1.3302)
	Lick rate (Hz): response	mCherry: 6 mice; ChR2: 4 mice; eNpHR: 4 mice	RM two-way ANOVA	Time x Group: $P = 0.6705$; Time: $P = 0.8115$; Group: $P = 0.3143$;	Time x Group: $F(2, 11) = 0.5216$; Time: $F(1, 11) = 0.0597$; Group: $F(2, 11) = 1.288$	mCherry Light on: 4.3667 (0.6227); mCherry Light off: 4.6500 (0.6339); ChR2 Light on: 4.3750 (0.7238); ChR2 Light off: 4.5750 (1.5184); eNpHR Light on: 6.1500 (0.3096); eNpHR Light off: 5.8500 (0.3069)	
	light on: mCherry vs. ChR2:		post hoc Bonferroni multiple comparisons	$P = 1$			-0.1617 (-2.7181, 2.1868)
	light on: mCherry vs. eNpHR:		post hoc Bonferroni multiple comparisons	$P = 0.3249$			-1.217 (-3.8673, -0.0423)
	light on: eNpHR vs. ChR2:		post hoc Bonferroni multiple comparisons	$P = 0.4725$			-1.1536 (-4.4726, 0.7179)
	light off: mCherry vs. ChR2:		post hoc Bonferroni multiple comparisons	$P = 1$			-0.0501 (-2.6298, 2.4652)
	light off: mCherry vs. eNpHR:		post hoc Bonferroni multiple comparisons	$P = 0.8164$			-0.7472 (-2.9493, 0.5157)
	light off: eNpHR vs. ChR2:		post hoc Bonferroni multiple comparisons	$P = 0.8592$			-0.4211 (-2.9808, 1.5991)
