# Supplementary material for: Anterior cingulate cortex provides the neural substrates for feedback-driven iteration of decision and value representation
Source: Nat Commun. 2024 Jul 17;15:6020. doi: 10.1038/s41467-024-50388-9 (PMC11255269; doi:10.1038/s41467-024-50388-9)
Supplement: Supplementary file 3 — Reporting Summary [file 41467_2024_50388_MOESM3_ESM.pdf]

nature portfolio

Corresponding author(s): Yunyun Han

Last updated by author(s): Jun 20, 2024

Reporting Summary

Nature Portfolio wishes to improve the reproducibility of the work that we publish. This form provides structure for consistency and transparency in reporting. For further information on Nature Portfolio policies, see our Editorial Policies and the Editorial Policy Checklist.

Statistics

For all statistical analyses, confirm that the following items are present in the figure legend, table legend, main text, or Methods section.

n/a	Confirmed	
☐	☒	The exact sample size (*n*) for each experimental group/condition, given as a discrete number and unit of measurement
☐	☒	A statement on whether measurements were taken from distinct samples or whether the same sample was measured repeatedly
☐	☒	The statistical test(s) used AND whether they are one- or two-sided<br>*Only common tests should be described solely by name; describe more complex techniques in the Methods section.*
☒	☐	A description of all covariates tested
☐	☒	A description of any assumptions or corrections, such as tests of normality and adjustment for multiple comparisons
☐	☒	A full description of the statistical parameters including central tendency (e.g. means) or other basic estimates (e.g. regression coefficient) AND variation (e.g. standard deviation) or associated estimates of uncertainty (e.g. confidence intervals)
☐	☒	For null hypothesis testing, the test statistic (e.g. *F*, *t*, *r*) with confidence intervals, effect sizes, degrees of freedom and *P* value noted<br>*Give P values as exact values whenever suitable.*
☒	☐	For Bayesian analysis, information on the choice of priors and Markov chain Monte Carlo settings
☒	☐	For hierarchical and complex designs, identification of the appropriate level for tests and full reporting of outcomes
☐	☒	Estimates of effect sizes (e.g. Cohen's *d*, Pearson's *r*), indicating how they were calculated

Our web collection on statistics for biologists contains articles on many of the points above.

Software and code

Policy information about availability of computer code

Data collection	Behavioral data were collected with ArControl software (https://github.com/chenxinfeng4/ArControl, Version 0.3.3.). Two-photon calcium imaging was performed with a resonant-galvo scanning two-photon microscope (Scientifica; 30 fps; 512 × 512 pixels) through a 16×/0.8NA waterimmersion objective (Nikon). Images were acquired by ScanImage software (Vidrio Technologies, Version 2017b)
Data analysis	ImageJ software (US National Institutes of Health, Version 1.54f) was used for extracting fluorescence traces from image stacks. Data analysis and statistical tests were performed with custom scripts written in MATLAB (MathWorks, 2020a). and Graphpad Prism (Version 9.1). All custom codes were available at https://github.com/ChenWq2023/ACC and https://zenodo.org/doi/10.5281/zenodo.12177591

For manuscripts utilizing custom algorithms or software that are central to the research but not yet described in published literature, software must be made available to editors and reviewers. We strongly encourage code deposition in a community repository (e.g. GitHub). See the Nature Portfolio guidelines for submitting code & software for further information.

Data

Policy information about availability of data

All manuscripts must include a data availability statement. This statement should provide the following information, where applicable:

- Accession codes, unique identifiers, or web links for publicly available datasets
- A description of any restrictions on data availability
- For clinical datasets or third party data, please ensure that the statement adheres to our policy

The data that support the findings of this study are available in figshare (https://figshare.com/s/eee94309c2b4297357fb). Source data are provided as a Source Data file.

Research involving human participants, their data, or biological material

Policy information about studies with human participants or human data. See also policy information about sex, gender (identity/presentation), and sexual orientation and race, ethnicity and racism.

Reporting on sex and gender	N/A
Reporting on race, ethnicity, or other socially relevant groupings	N/A
Population characteristics	N/A
Recruitment	N/A
Ethics oversight	N/A

Note that full information on the approval of the study protocol must also be provided in the manuscript.

Field-specific reporting

Please select the one below that is the best fit for your research. If you are not sure, read the appropriate sections before making your selection.

☒ Life sciences ☐ Behavioural & social sciences ☐ Ecological, evolutionary & environmental sciences

For a reference copy of the document with all sections, see nature.com/documents/nr-reporting-summary-flat.pdf

Life sciences study design

All studies must disclose on these points even when the disclosure is negative.

Sample size	No statistical methods were used to predetermine sample sizes, but our study used similar sample sizes as previous studies (PMID: 32884146;PMID: 32868778;PMID: 33243980)
Data exclusions	No data exclusions. One mouse (56 neurons recorded) was not recorded for the Reversal and Re-stable sessions due to the infection of craniotomy.
Replication	All experiments and analysis were repeated for at least four subjects. All attempts at replication were successful.
Randomization	Mice were randomly assigned to different experimental groups; Stimuli were randomly interleaved during tasks.
Blinding	Two-photon calcium imaging data collection and analysis were not performed in a blinded fashion as only one cohort was record. The investigators were blinded to the experimental groups during optogenetic experiment.

Reporting for specific materials, systems and methods

We require information from authors about some types of materials, experimental systems and methods used in many studies. Here, indicate whether each material, system or method listed is relevant to your study. If you are not sure if a list item applies to your research, read the appropriate section before selecting a response.

Materials & experimental systems

n/a	Involved in the study	
☒	☐	Antibodies
☒	☐	Eukaryotic cell lines
☒	☐	Palaeontology and archaeology
☐	☒	Animals and other organisms
☒	☐	Clinical data
☒	☐	Dual use research of concern
☒	☐	Plants

Methods

n/a	Involved in the study	
☒	☐	ChIP-seq
☒	☐	Flow cytometry
☒	☐	MRI-based neuroimaging

Animals and other research organisms

Policy information about studies involving animals; ARRIVE guidelines recommended for reporting animal research, and Sex and Gender in Research

Laboratory animals	C57BL/6J male mice from Vital River (2-4 months old, 20-30 g) were used in this study. The animals were singly housed under under a constant temperature (25 ± 1 °C), humidity (40–60%) and 12-h light/dark cycle (8:00 a.m. to 20:00 p.m.).
Wild animals	Wild animals were not used in this study
Reporting on sex	Only male mice were used in this study
Field-collected samples	Field-collected samples were not used in this study.
Ethics oversight	All procedures were performed following protocols approved by the Animal Care and Use Committee at Huazhong University of Science and Technology

Note that full information on the approval of the study protocol must also be provided in the manuscript.

Plants

Seed stocks	N/A
Novel plant genotypes	N/A
Authentication	N/A
